# Reference‐Guided Chromosome‐by‐Chromosome *de novo* Assembly at Scale Using Low‐Coverage High‐Fidelity Long‐Reads with HiFiCCL

**DOI:** 10.1002/advs.202515308

**Published:** 2025-12-25

**Authors:** Zhongjun Jiang, Weihua Pan, Runtian Gao, Heng Hu, Wentao Gao, Murong Zhou, Yu‐Hang Yin, Zhipeng Qian, Shuilin Jin, Guohua Wang

**Affiliations:** ^1^ College of Life Science Northeast Forestry University Harbin China; ^2^ College of Computer and Control Engineering Northeast Forestry University Harbin China; ^3^ State Key Laboratory of Genome and Multi‐Omics Technologies, Shenzhen Branch Guangdong Laboratory for Lingnan Modern Agriculture Agricultural Genomics Institute at Shenzhen Genome Analysis Laboratory of the Ministry of Agriculture and Rural Affairs Chinese Academy of Agricultural Sciences Shenzhen China; ^4^ School of Mathematics Harbin Institute of Technology Harbin China; ^5^ School of Computer Science and Technology Harbin Institute of Technology Harbin China

**Keywords:** chromosome‐by‐chromosome, low coverage, long high‐fidelity reads, population genomics, reference‐guided de novo assembly

## Abstract

Population genomics using short‐read resequencing captures single‐nucleotide polymorphisms and small insertions and deletions but struggles with structural variants, leading to a loss of heritability in genome‐wide association studies. In recent years, long‐read sequencing has improved pangenome construction for diverse eukaryotic species, including humans, crops, and other organisms of ecological and economic importance, addressing this issue to some extent. Sufficient‐coverage high‐fidelity data for population genomics is often prohibitively expensive, limiting its use in large‐scale populations and broader eukaryotic species and creating an urgent need for robust low‐coverage assemblies. However, current assemblers underperform in such conditions. To address this, HiFiCCL is proposed, the first assembly framework specifically designed for low‐coverage high‐fidelity reads, using a reference‐guided, chromosome‐by‐chromosome assembly approach. This study demonstrates that HiFiCCL improves low‐coverage assembly performance of existing assemblers and outperforms the state‐of‐the‐art assemblers on human and plant datasets. Tested on 45 human datasets (∼5× coverage), HiFiCCL combined with hifiasm reduces the length of misassembled contigs relative to hifiasm by an average of 21.19% and up to 38.58%. These improved assemblies excel in detecting large germline structural variants, minimize inter‐chromosome mis‐scaffolding, and improve the detection of specific germline and tumor somatic structural variants based on the pangenome graph.

## Graphical abstract

HiFiCCL, as the first assembly framework specifically designed for low‐coverage high‐fidelity reads, improves the assembly quality of existing assemblers and also enhances downstream applications such as large structural variant (SV) detection (>10 000 bp), synteny analysis, pangenome graph construction, and graph‐based individual‐specific germline SVs and tumor somatic SVs detection, thereby advancing the field of population genomics.

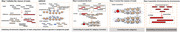

## 1 Introduction

Population genomics is crucial for understanding genetic variation within and between populations, shedding light on evolutionary processes, adaptation, and the genetic basis of traits. It has broad applications in areas such as human health, agriculture, and disease management [1, 2]. Due to the limitations of read length, the population genomes based on short‐read resequencing data primarily capture information on single‐nucleotide polymorphisms (SNPs) and small insertions and deletions (indels) but struggle to accurately detect structural variants (SVs), leading to a loss of heritability in genome‐wide‐association studies (GWAS) [3, 4, 5]. To overcome this limitation, recent efforts have led to the construction of pangenomes for a large number of eukaryotic species (e.g., human [6, 7], tomato [8], rice [9]), featuring high quality, with accurate and complete SV detection, pangenomes can significantly improve heritability compared to resequencing‐based populations [10, 11, 12]. However, despite a decrease in cost over the years, long‐read sequencing remains significantly more expensive than short‐read sequencing, limiting its application in building large‐scale populations (thousands of individuals or more) and in broader eukaryotic species. Therefore, there is an urgent need for methodological innovations to achieve population‐scale genome assemblies of high quality at a cost that is competitive and feasible, depending on the sequencing platform and study goals.

One possible approach to achieve this goal is to assemble low‐coverage (defined here as 5×–10×) long reads. This strategy produces a pair of assemblies: one primary assembly, which represents a mosaic of homologous haplotypes, and one alternate assembly, composed of short haplotype‐specific contigs (haplotigs) that capture alleles absent from the primary assembly. Compared to standard short‐read resequencing (typically 30×–60×), this approach is more cost‐effective for applications such as structural variation (SV) detection, *de novo* genome assembly, and resolving complex or repetitive genomic regions. Based on the type of data used, existing long‐read assembly algorithms can be classified into: 1) algorithms based on noisy reads (ONT or PacBio CLR), 2) algorithms based on high‐fidelity (PacBio HiFi) reads, and 3) algorithms based on hybrid data (both noisy reads and HiFi reads) [13]. The noisy‐read‐based assemblers (e.g., Canu [14], Flye [15], wtdbg [16], Nextdenovo [17], MECAT [18], Hifiasm (ONT) [19]) focus on addressing sequencing errors in reads to minimize their impact on the accuracy and continuity of the assembly. The HiFi‐based assemblers (e.g., Hifiasm [20, 21], HiFlye [15], LJA [22]) focus on leveraging the dual advantages of HiFi reads in accuracy and length to achieve assembly goals, such as chromosome haplotyping and the assembly of complex regions. The hybrid assemblers (e.g., Hifiasm (UL) [23], Verkko [24]) integrate the ONT ultra‐long reads on the HiFi‐based assembly graph to solve the ultra‐complex genomic regions and can generate chromosome‐level contigs in some situations. However, nearly all these assembly methods are primarily developed for long reads with high coverage (typically greater than 30×), and may encounter challenges under low‐coverage sequencing conditions, such as increased assembly errors (e.g., misassemblies (MA) of similar sequences between chromosomes due to insufficient coverage) and reduced genome completeness.

A potential solution is to use a reference genome to alleviate the challenges of assembling low‐coverage data. After all, when attempting to construct a population genome for a species, there is typically at least one high‐quality reference genome available. Furthermore, genomics has entered the telomere‐to‐telomere (T2T) era, with an increasing number of high‐quality reference genomes being released for various species [13, 25, 26, 27, 28, 29]. This makes such as approach applicable to a much broader range of scenarios. In previous related studies, TRFill [30] effectively addressed the haplotype‐resolved assembly of long tandem repeats by using a high‐quality reference genome to bin reads from different complex genomic regions. GALA [31] optionally utilizes a reference genome to assist multiple preliminary assemblies in guiding reads to cluster by chromosomes, enabling chromosome‐by‐chromosome assembly. Notably, these methods, collectively referred to as reference‐guided *de novo* assembly, differ from traditional reference‐based assembly methods [32]. Instead of directly assembling reads based on their alignment positions on the reference genome, they only use reference to classify the reads of different genomic regions and then perform *de novo* assembly. This approach not only leverages the reference genome to reduce the difficulty of assembly but also enables accurate assembly of genomic regions with large SVs. However, despite these studies demonstrating the effectiveness of reference‐guided *de novo* assembly on high coverage long‐reads data, their performance remains suboptimal under low‐coverage conditions.

In this paper, we propose HiFiCCL, the first assembly framework specifically designed for low‐coverage HiFi reads, using an exclusively reference‐guided, chromosome‐by‐chromosome *de novo* assembly approach. We validate the effectiveness of HiFiCCL across various applications, including contig‐level assembly, large germline SVs detection (larger than 10,000 bp), chromosome‐level scaffolding, construction of pangenome graph and pangenome‐graph‐based large germline SVs and tumor somatic SVs detection.

## 2 Result

### 2.1 Overview of the HiFiCCL framework

**FIGURE 1:**
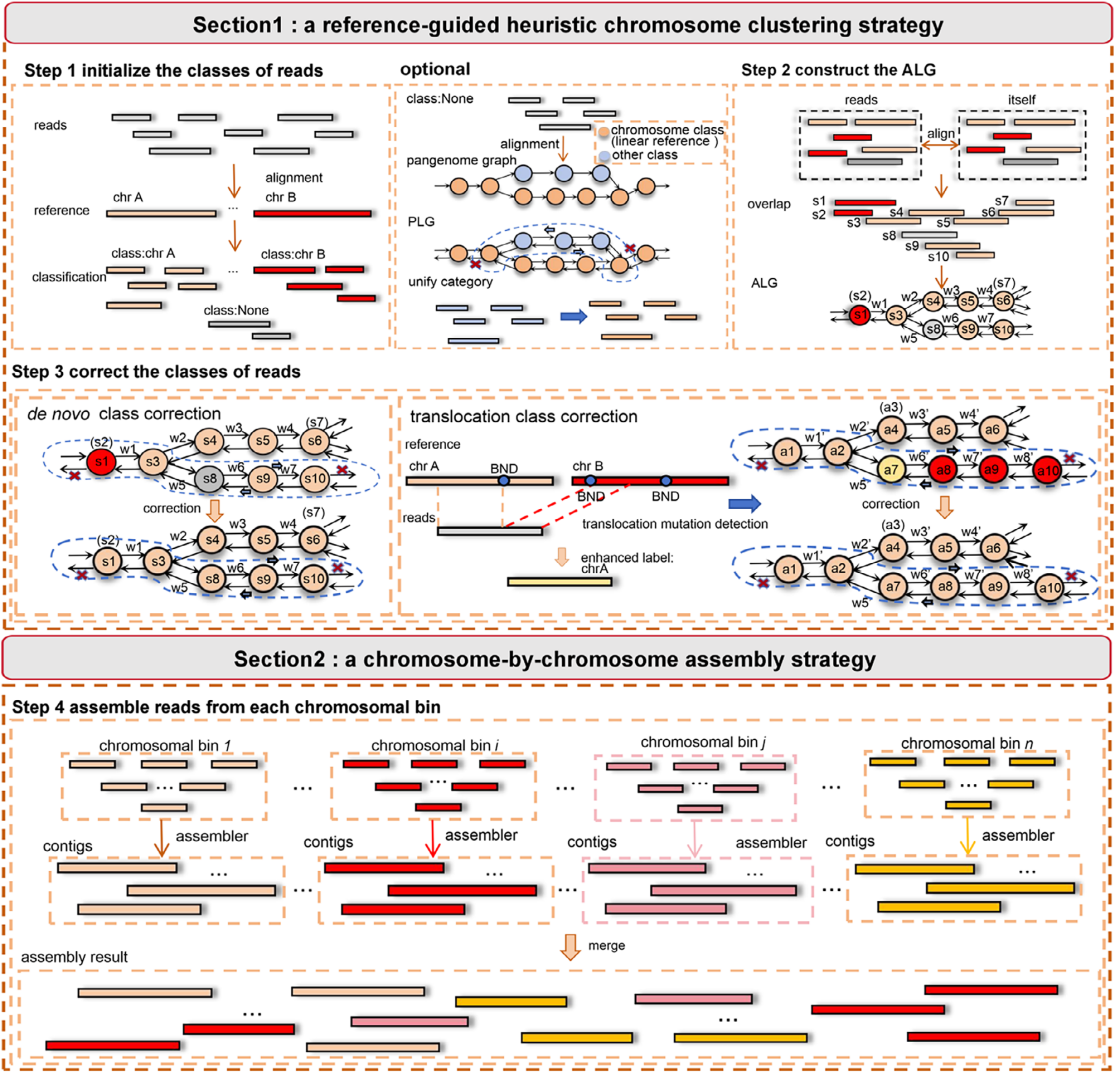
Overview of the HiFiCCL framework workflow. HiFiCCL is structured into two sections. Section 1 encompasses three steps: initially, reads are aligned with the linear reference genome, and chromosomal classes for reads are initialized based on this alignment information. In this process, reads lacking chromosomal class information are classified as “None,” depicted in gray, while reads with distinct chromosomal classes are represented in other colors. An optional mode of HiFiCCL involves aligning reads labeled as “None” to the pangenome graph. These reads are then assigned chromosomal classes by searching through the constructed Pangenome Label Graph (PLG) (see the Experimental Section). The second step involves inter‐read alignments from which the proposed Alignment Label Graph (ALG) (see the Experimental Section) is constructed. The third step utilizes the ALG generated from the preceding steps to correct class errors and complete the chromosome clustering process. The search begins by randomly selecting a node (e.g., node “s9”) and proceeds bidirectionally using a greedy approach to select the node with the highest overlap degree, until a significant drop in overlap degree is encountered (see the Experimental Section). This identifies a path that serves as evidence for *de novo* class correction. For translocation mutations, class correction incorporates alignment information and a translocation detection algorithm to identify breakpoints. Reads containing breakpoints information are corrected to include enhanced classes. If an enhanced class is encountered during path searching, all reads on that path are updated to reflect enhanced chromosomal information. Section 2 then follows, where reads within each chromosomal cluster are assembled using an existing assembler, and then contigs from all chromosomal bins are subsequently merged to yield the final assembly.

Inspired by GALA, HiFiCCL designs a novel exclusively referenced‐guided chromosome‐by‐chromosome *de novo* assembly strategy, where whole‐genome reads are partitioned by chromosome using the high‐quality reference genome and then assembled individually. Under low sequencing coverage, the lack of sufficient read depth increases the likelihood of reads with overlapping relations between different chromosomes being misassembled, leading to assembly errors. By preventing interference between sequences from similar regions on different chromosomes, this approach reduces assembly difficulty and improves overall assembly quality. The key challenge of chromosome‐by‐chromosome assembly lies in accurately clustering reads by chromosome. To achieve this, GALA combines multiple sets of preliminary assemblies to guide the clustering process and can optionally incorporate a reference genome as an auxiliary aid. However, in the case of low sequencing coverage, the quality of preliminary assemblies drops dramatically, with high assembly errors and incomplete genome sizes, making it difficult to accurately cluster the reads. To address this, HiFiCCL uses either a high‐quality linear reference genome (default mode) or a combination of the linear reference genome and a pangenome graph (optional mode) to guide the chromosome‐based classification of reads. It incorporates a series of heuristic strategies to improve the accuracy of read clustering and reduce MA caused by ambiguous overlaps between sequences from different chromosomes under low sequencing coverage (Figure 1 and Figure S1).

First, each read is aligned to the linear reference genome and assigned to all potential chromosome categories. However, due to sequence differences between the reference genome and the genome to be assembled, alignment errors, and highly similar regions between chromosomes, a certain proportion of reads may remain unclassified or misclassified. HiFiCCL optionally utilizes a pangenome graph, which incorporates the majority of variant sequences of the species and uses a linear reference genome as the backbone, to mitigate the impact of unclassified reads. Specifically, unclassified reads are aligned to the pangenome graph, and those that align to the linear reference regions are directly assigned to their initial categories based on chromosome IDs. For the reads that align to bubble regions in the pangenome graph, it is difficult to directly determine their classification information due to the lack of chromosome categories in these regions. HiFiCCL addresses this issue by leveraging the pangenome graph structure, assuming that bubble regions with high sequence cohesion to a specific chromosome in the linear reference genome also belong to that chromosome. Specifically, a Depth‐First‐Search (DFS)‐based graph traversal algorithm is used, starting from the alignment position of the read in the graph, and performing a bidirectional search until a position in the linear reference genome is reached.

Second, *de novo* class correction is introduced as a key strategy to address the misclassification and unclassification of reads by leveraging alignment information between reads to identify reliably overlapping reads and using their chromosome classes for voting‐based correction. Specifically, HiFi reads from the genome to be assembled are used to construct a labeled bidirectional overlap graph, referred to as the ALG, where each node represents a read, edges represent overlaps, and edge weights indicate the degree of overlap (as defined in the Experimental Section, reflecting the reliability of the connection). Each node is labeled with the chromosome ID to which the read belongs. By starting bidirectional searches from randomly selected nodes, the search is truncated when a significant drop in edge weight is encountered (see the Experimental Section). This process partitions the overlap graph into reliable linear subgraphs, where all nodes within each subgraph are determined to belong to the same chromosome.

Due to translocation variations between the reference genome and the genome being assembled, reference‐based clustering may incorrectly assign reads from translocated genomic regions to the wrong chromosomes. When the translocated region is substantial, the aforementioned correction method may result in a scenario where the number of misclassified reads within a subgraph surpasses that of correctly classified reads, ultimately leading to erroneous corrections. To address this issue, HiFiCCL employs the translocation variation detection algorithm to call translocation variations and the breakpoints. Based on this, it assigns the reads used for breakpoint identification to their correct chromosome IDs. Then, in the overlap graph, during the search process, these reads are encountered, and all reads within the corresponding subgraph are assigned to the same chromosome class as the breakpoint reads.

To ensure robustness and mitigate potential biases introduced by the random selection of starting nodes during graph traversal, the process is repeated *M* times with randomized node orders. For each iteration, the chromosome of all nodes (reads) within each subgraph is determined through a voting mechanism. After *M* iterations, the final class for each node is assigned based on the majority vote across all iterations.

Finally, *de novo* assembly is performed for reads within each chromosomal cluster based on their overlap relationships, aiming to reduce the risk of MA caused by potential misclassification of reads across chromosomes.

### 2.2 Performance on human datasets

We evaluated the default mode of HiFiCCL against other state‐of‐the‐art assemblers, including Hifiasm, HiFlye, LJA, Verkko, and GALA, on the HG002 and NA19240 HiFi datasets at approximately 5× coverage. For HiFiCCL, Hifiasm was selected as the base assembler, and CHM13 2.0 [27] was used as the reference genome. It is worth noting that high‐quality reference genomes corresponding to the test datasets were used to assess assembly accuracy. The results (Table 1, Table S1) demonstrated that the assemblies produced by HiFiCCL‐Hifiasm, Hifiasm, and HiFlye closely approximated the true genome size, whereas those generated by LJA and Verkko were markedly smaller relative to the actual genome size. Among the three methods that produced relatively complete genomes, HiFiCCL‐Hifiasm demonstrated comparable levels of contiguity, with a slight increase in BUSCO [34] gene completeness and Merqury [35] completeness compared to Hifiasm, while exhibiting the lowest misassembly contigs length (MCL), which was significantly smaller than that of Hifiasm. For example, for the HG002 dataset, HiFiCCL‐Hifiasm reduced the MCL by 17.38% (from 331.84 to 274.16 Mb), and for the NA19240 dataset, the reduction was even greater, reaching 27.16% (from 245.96 to 179.15 Mb). These results highlight HiFiCCL‐Hifiasm's ability to effectively reduce assembly errors while maintaining high levels of genome assembly completeness and quality. In addition, it achieved a significantly faster runtime compared to Hifiasm (Table S2), showing that HiFiCCL‐Hifiasm not only improves assembly quality but also substantially accelerates the process by more than 1.5 times on low‐coverage (∼5×) human datasets, under comparable computational resources and memory usage relative to Hifiasm. It is important to note that we tested GALA with it supported all assemblers, using a linear reference genome and three assembly results as input. However, at low coverage, the input results from other assemblers (such as Hifiasm, HiFlye, and LJA) generally exhibited lower contiguity and higher assembly error rates. As a result, GALA tended to over‐partition the read sequences into far more bins than the actual number of chromosomes, ultimately leading to assembly failures. In addition, GALA's complexity, particularly the chromosomal clustering step which took over 20 times longer than HiFiCCL (Table S3), may contribute to its unsuitability for low‐coverage assembly scenarios. Furthermore, we tested HiFiCCL and other assemblers on two plant datasets [58, 59, 60], as detailed in Note S1.

**TABLE 1:** Statistics of human primary assemblies across different assemblers. HiFiCCL‐Hifiasm indicates the use of Hifiasm as the base assembler for HiFiCCL. The default mode of HiFiCCL was employed, which uses only CHM13 2.0 [27] as the reference genome for guidance. MCL, which stands for “misassembled contigs length,” is a metric used by the Quast [33] tool to evaluate the assembly accuracy by comparing assembled genomes against the corresponding high‐quality genome. The NG50 of an assembly is defined as the sequence length of the shortest contig at 50% of the total genome size. The NGA50 of an assembly is the length of the correctly aligned block at 50% of the total reference genome size, which is assumed to be 3.1 Gb. “Complete” refers to the percentage of all complete single‐copy and duplicated orthologous genes. “Single” refers to the percentage of all complete orthologous genes that exist in a single copy.

Dataset	Assembler	Size (Gb)	Contigs number	MCL (Mb)	NG50 (kb)	NGA50 (kb)	Gene completeness (BUSCO)	
							Complete /Single(%)	Missing (%)
HG002 (HiFi 5x)	HiFiCCL‐Hifiasm	2.79	18 304	274.16	199.05	185.04	82.6/79.1	7.1
	Hifiasm	2.73	17 868	331.84	194.55	174.55	82.0/78.7	7.6
	HiFlye	3.35	39 567	501.71	199.45	175.02	84.3/76.9	6.1
	LJA	1.7	11 913	211.1	83.1	70.5	47.6/45.2	43.0
	Verkko	2.0	43 952	49.0	31.4	29.5	42.3/36.9	44.8
	GALA	—	—	—	—	—	—	—
NA19240 (HiFi 5x)	HiFiCCL‐Hifiasm	2.64	23 417	179.15	124.83	116.19	77.9/74.9	9.9
	Hifiasm	2.57	22 561	245.96	123.48	112.86	77.5/74.5	10.1
	HiFlye	3.06	43 236	259.01	134.33	125.70	77.4/70.8	10.3
	LJA	0.64	4955	115.58	—	—	22.2/21.8	72.1
	Verkko	1.06	24 974	27.42	—	—	26.7/23.2	63.1
	GALA	—	—	—	—	—	—	—

The influence of varying coverage levels and different base assemblers on the proposed HiFiCCL was evaluated across multiple coverage levels of the HG002 dataset (∼3×, 5×, 8×, and 11×), using Hifiasm, HiFlye, and LJA to assess their impact on assembly performance (Tables S7 and S8). HiFiCCL was applied in two modes: the default mode, which used CHM13‐T2T as a linear reference for clustering guidance, and the optional mode, which required both CHM13‐T2T reference genome and a human pan‐genome reference [6] constructed using minigraph [36] (hprc‐v1.0‐minigraph‐chm13.gfa). The pangenome graph included 45 human genomes but excludes the HG002 dataset being analyzed. HiFiCCL showed the most significant improvements at 5× coverage, particularly in reducing MCL. For instance, HiFiCCL‐Hifiasm (optional mode) reduced MCL by 17.40% (from 331.84 to 274.07 Mb) and improved contiguity metrics, while HiFiCCL‐HiFlye (optional mode) decreased MCL from 501.71 to 416.26 Mb. LJA, which struggled at low coverage, achieved the most notable gains at 5×, with HiFiCCL (primary mode) reducing MCL by over 50% (from 211.11 to 100.10 Mb) and delivering comparable contiguity metrics despite smaller genome sizes. At 8x, HiFiCCL‐LJA (optional mode) achieved the lowest MCL (from 120.08 to 90.60 Mb). As coverage increased, their contiguity metrics significantly improved. Compared to the default mode, the optional mode of HiFiCCL provided only marginal benefits because it merely extends the default mode by aligning reads that fail to map to the linear reference genome against a pangenome graph to assign initial labels. These labels are subsequently refined through a *de novo* correction strategy, which effectively reduces the proportion of unclassified reads (Figure S2). By incorporating information from the pangenome graph, the optional mode enhances classification inference, though its impact remains limited. We further evaluated the performance of HiFiCCL's optional mode using human pangenome graphs constructed with different tools such as Minigraph [36], Minigraph‐Cactus [37] (based on linear references like CHM13 or GRCh38) (see Note S2).

To evaluate the impact of linear reference genome selection on HiFiCCL assembly in its default mode, we tested the CHM13 dataset (about 5× coverage) using various reference genomes, including CHM13 2.0, HG002_maternal, HG002_paternal, GRCH37, GRCH38, and more distantly related species such as Chimpanzee and Siamang [38]. Using HiFiCCL in its default mode with Hifiasm as the base assembler, as shown in Table 2, reference‐guided *de novo* assembly significantly outperformed traditional reference‐free assembly in genome completeness and accuracy. Under optimal conditions (CHM13 2.0 as the reference and CHM13 as the test dataset), HiFiCCL‐Hifiasm reduced MCL by 31.59% (from 291.86 to 199.64 Mb), decreased erroneous translocations from 562 to 120, increased NG50 by 11.28% (from 169.59 to 188.73 kb), improved BUSCO completeness from 78.4% to 82.7%, and increased merqury completeness from 83.81% to 90.64% (Table S9). Similar improvements were observed with the use of other human reference genomes. For example, using HG002_paternal as the reference genome, despite lacking the X chromosome, still outperformed Hifiasm alone. Using Chimpanzee or Siamang as reference genomes, the number of erroneous translocations increased compared to human references but remained substantially lower than reference‐free assembly. Other metrics, such as MCL, BUSCO, and merqury completeness, also improved. Notably, with Siamang as the reference, MCL were reduced to 119.31 Mb, demonstrating that the choice of reference genome has limited impact on HiFiCCL performance on human datasets. Closer evolutionary relationships generally resulted in better assembly outcomes (with the lower translocation metric). Further analysis of Siamang‐guided HiFiCCL‐Hifiasm assemblies in the MHC region (e.g., HLA‐A, HLA‐B, HLA‐C, HLA‐DQA1, HLA‐DQB1, and HLA‐DRB1) showed strong reconstruction performance (Table S10). Unless otherwise specified, HiFiCCL refers to its default mode of operation in the following sections.

**TABLE 2:** Performance of HiFiCCL‐Hifiasm (default mode) using different reference genomes on the CHM13 dataset. In the “Reference” column, a dash (“‐”) indicates that the assembly was performed using only Hifiasm, while other genome names represent the default mode of HiFiCCL‐Hifiasm, where these reference genomes were used to guide the binning process. The translocation metric denotes the number of erroneous translocations identified in comparison with the CHM13 2.0 reference genome.

Dataset	Reference	Size (Gb)	Contigs number	MCL(Mb)/ translocation	NG50 (kb)	NGA50 (kb)	Gene completeness (busco)	
							Complete /Single(%)	Missing (%)
CHM13 (HiFi 5×)	—	2.48	15 684	291.86/562	169.59	159.77	78.4/75.9	11.4
	CHM13 2.0	2.70	16 926	199.64/120	188.73	181.96	82.7/79.9	7.6
	HG002_maternal	2.70	16 976	201.86/148	188.26	181.34	82.6/79.9	7.7
	HG002_paternal	2.67	18 314	199.36/147	178.10	171.29	82.2/79.4	8.0
	GRCH37	2.74	17 340	207.87/141	189.82	183.58	82.6/79.9	7.4
	GRCH38	2.69	17 177	184.41/152	185.74	179.83	82.7/79.8	7.6
	Chimpanzee	2.70	17 860	184.25/181	181.27	175.52	82.6/79.8	7.5
	Siamang	2.60	17 489	119.31/225	172.06	168.94	82.6/79.9	7.6

### 2.3 Benchmarking SV detection on HG002 dataset

To evaluate the potential of assemblies from HiFiCCL in improving SVs detection, we conducted SVs calling using SVIM‐asm [41] on the assemblies generated by HiFiCCL‐Hifiasm and Hifiasm at ∼5× coverage, as well as HiFiCCL‐HiFlye, HiFlye, HiFiCCL‐LJA, and LJA at ∼8× coverage. The SVs calling was benchmarked against SVs within the high‐confidence regions as defined by the HG002 GIAB Tier 1 dataset [39], which is a widely recognized and classic benchmark extensively used for evaluating SV detection methods. The results (Figure 3A, Table S11) further validated previous findings and demonstrated that HiFiCCL improved the performance of base assemblers, as shown by improved structural variant detection capabilities.

**FIGURE 2:**
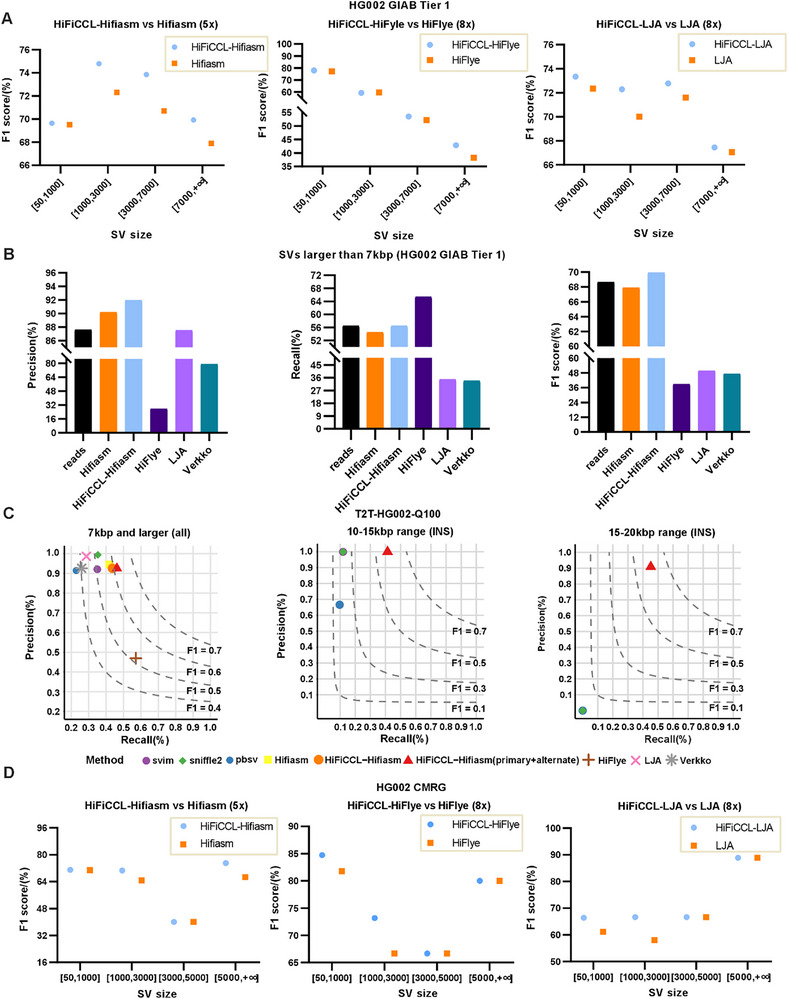
SV detection and comparison on the HG002 dataset. (A) Comparison of F1 scores for SV detection (GIAB Tier 1 [39]) based on assemblies from HiFiCCL‐Hifiasm and Hifiasm, HiFiCCL‐HiFlye and HiFlye, and HiFiCCL‐LJA and LJA across different SV sizes. (B) Comparison of large SVs (>7000 bp) detection from various assemblies with the reads‐alignment‐based SVs calling, where “reads” indicate SVs detected through reads alignment‐calling method (SVIM [40]), while different assemblers represent SVs identified from their respective assemblies (SVIM‐asm [41]). (C). Performance of HiFiCCL‐Hifiasm (primary+alternate) in large SV detection compared to read‐based tools. HiFiCCL‐Hifiasm (primary+alternate) utilizes both primary assembly results and alternate assembly results for SV detection, and its performance is compared with three read‐based SV detection tools: svim, sniffles2 [42], and pbsv (https://github.com/PacificBiosciences/pbsv). “INS” indicates testing on insertion‐type SV. (D) Comparison of SV detection in the CMRG [43] region based on assemblies from HiFiCCL‐Hifiasm, Hifiasm, HiFiCCL‐HiFlye, HiFlye, HiFiCCL‐LJA, and LJA across different SVs sizes.

Assembly‐based SVs detection generally facilitates the identification of larger SVs compared to alignment‐based SVs detection [44]. To assess whether assemblies enhance the detection of large SVs, we selected SVIM [40] and SVIM‐asm [41], two tools based on similar heuristic rules, using the HG002 dataset at 5× coverage. SVIM represents a read‐alignment‐based approach, while SVIM‐asm utilizes alignments from various assemblies (e.g., Hifiasm, HiFiCCL‐Hifiasm, HiFlye, LJA, Verkko). The size distribution of SVs was shown in Figure S3, where it was evident that assembly‐based SVs detection identified more SVs larger than 7000 bp compared to read‐based SVs detection. We further compared detection performance for SVs larger than 7000 bp (Figure 2B, Table S12). The results indicated that HiFiCCL‐Hifiasm exhibited higher precision (0.9193) and a recall rate (0.5643) comparable to that of the “reads” category (where “reads” is used to denote the reads‐alignment‐based method for detecting SVs), along with the highest F1 score (0.6993).

To extend this evaluation, we utilized the latest high‐resolution Genome in a Bottle (GIAB) SV benchmark dataset, based on the T2T‐HG002‐Q100 diploid assembly (https://ftp‐trace.ncbi.nlm.nih.gov/ReferenceSamples/giab/data/AshkenazimTrio/analysis/NIST_HG002_DraftBenchmark_defrabbV0.019‐20241113/). This updated benchmark includes ∼29 167 confident SVs, three times more than the previous version, enabling a more comprehensive and detailed assessment of SV detection performance across calling tools and assembly methods. Both read‐based (SVIM, pbsv, sniffles2) and assembly‐based (SVIM‐asm with Hifiasm, HiFiCCL‐Hifiasm, HiFlye, LJA, Verkko) methods were tested (Figure 2C, Table S13), focusing on large SVs (>7000 bp). Assembly‐based methods achieved significantly higher recall for large SVs compared to the best‐performing read‐based tool (sniffles2, recall: 0.3526). For example, SVIM‐asm with HiFiCCL‐Hifiasm improved recall by 23.90% (0.4369 vs. 0.3526) while maintaining high precision (0.9255), resulting in the highest F1 score (0.6165) when combining primary and alternate assemblies (HiFiCCL‐Hifiasm (primary+alternate)). For insertions larger than 10 000 bp (Figure 2C, Table S14), assembly‐based methods excelled, with HiFiCCL‐Hifiasm (primary+alternate) achieving a precision of 0.9992 and F1 score of 0.5851 for insertions between 10 000 and 15 000 bp, compared to an F1 score of just 0.2136 for sniffles2. Notably, for insertions between 15 000 and 20 000 bp, only assembly‐based methods were effective, with HiFiCCL‐Hifiasm (primary+alternate) achieving a precision of 0.9084 and an F1 score of 0.6038. These results highlight the substantial advantages of assembly‐based SV detection, particularly for large variants, where they greatly improve recall and maintain high precision, underscoring the critical role of HiFiCCL‐Hifiasm in advancing SV detection accuracy.

We further evaluated the ability of HiFiCCL's assemblies in detecting challenging, medically relevant SVs (HG002 CMRG) [43], yielding promising results. The results (Figure 2D, Table S15) demonstrated that our method improved the detection of medically relevant SVs compared to the results from base assemblers. The improved detection of medically relevant SVs suggests that HiFiCCL produces more accurate and complete genome assemblies, preserving critical genomic regions that are often challenging to resolve. In addition, based on the commendable performance of HiFiCCL‐Hifiasm, Hifiasm, and reads‐alignment‐based detection (“reads”) in detecting SVs using the HG002 dataset (5×) within the GIAB Tier 1 benchmarks, we selected these three methods along with sniffles2 for further evaluation of their efficacy in detecting large, medically relevant SVs (greater than 5000 bp) (Table S16). The results indicated that HiFiCCL‐Hifiasm and sniffles2 also performed the best, achieving an accuracy of 1, despite the limited number of SVs in the CMRG set.

### 2.4 Performance of scaffolding and synteny analysis on human datasets

We scaffolded the assemblies of HiFiCCL‐Hifiasm, Hifiasm, HiFlye, LJA, Verkko, and GALA from the previously mentioned HG002 (5×) and NA19240 (5×) datasets using Ragtag [32]. The results (Table S17) demonstrated that only the scaffolds from HiFiCCL‐Hifiasm, Hifiasm, and HiFlye yielded more complete genomes, echoing previous findings. Among these, HiFiCCL‐Hifiasm exhibited the lowest number of MA and the highest completeness of single‐copy genes, with other metrics being comparable. This further underscores that our method improves the assembly quality of base assemblers, thereby improving scaffolding performance. After filtering out scaffolding sequences shorter than 5 Mb, our method continued to exhibit the lowest number of MA at the chromosomal level (Table S18). To assess HiFiCCL's ability to reconstruct complex rearrangements, we analyzed homozygous inversion SVs in chromosome 1 (chr1) of the HG002 sample. Using SyRI [45], we compared inversion detection results from HiFiCCL‐Hifiasm and other assemblers against GRCh38's chr1, leveraging the comprehensive inversion map [46] as a benchmark resource. HiFiCCL‐Hifiasm and Hifiasm identified 62.5% of inversions, outperforming HiFlye (50%), LJA (25%), and Verkko (12.5%) (Table S19).

**FIGURE 3:**
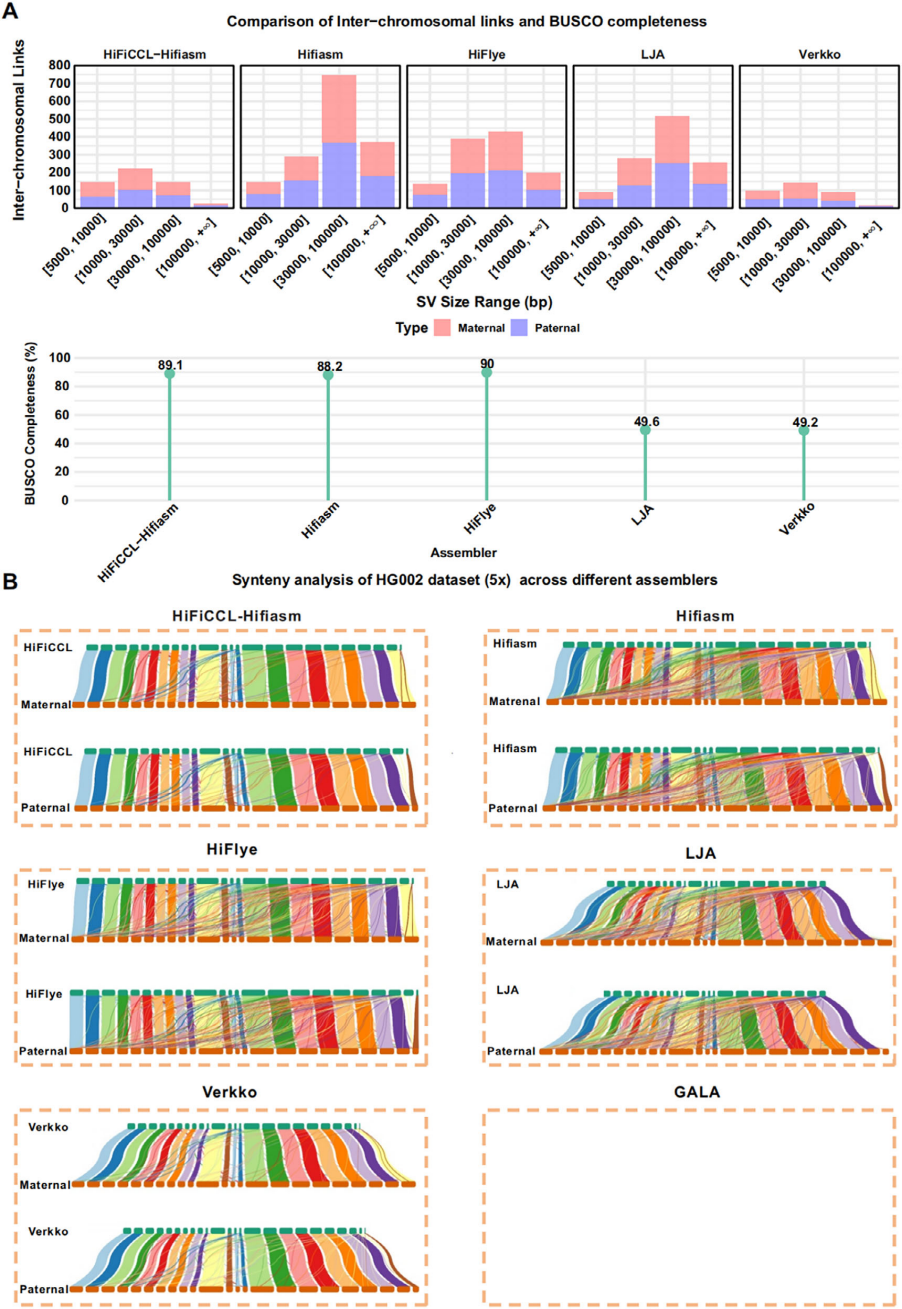
Synteny analysis of scaffolding results on low‐coverage HiFi dataset for HG002. (A) Comparative synteny analysis of different assemblers aligned to the reference genomes of HG002 maternal and HG002 paternal was performed, with a primary focus on the number of inter‐chromosomal links (considered as potential erroneous translocations). In addition, the BUSCO completeness scores of assemblies generated by different assemblers were also evaluated. (B) It shows the synteny between the scaffolding performance of different assemblies and the maternal and paternal reference genomes for HG002. Within each box, the top panel represents the synteny analysis with the maternal reference genome, while the bottom panel represents the synteny analysis with the paternal reference genome.

We then conducted a synteny analysis of these scaffolds (Figure 3, Figure S4) using the NGenomeSyn tool [47], aligning them to their corresponding high‐quality reference genomes. During this step, both inter‐ and intra‐chromosomal links shorter than 5000 bp were filtered to focus on meaningful SVs. In the analysis, inter‐chromosomal links were interpreted as potential translocations. The chromosome‐level scaffolds of HiFiCCL‐Hifiasm post‐scaffolding exhibited the best performance, with inter‐chromosomal mis‐scaffolding significantly reduced compared to Hifiasm post‐scaffolding (Figure 3A, Figure S4A, Table S20). For example, in the synteny analysis with the HG002 maternal reference genome, HiFiCCL‐Hifiasm had only 14 inter‐chromosomal links in regions >100 000 bp, compared to 189 links in the Hifiasm assembly, representing a 92.59% reduction. A similar trend was observed in the NA19240 dataset, with HiFiCCL‐Hifiasm consistently achieving fewer mis‐scaffolding events (Table S20). Figure 3B and Figure S4B provide a clear visualization of how HiFiCCL effectively reduces inter‐chromosomal mis‐scaffolding, outperforming all other assemblers in comparison. In addition, HiFiCCL‐Hifiasm achieved a much higher BUSCO completeness score compared to both Hifiasm and Verkko, while maintaining synteny and structural accuracy comparable to Verkko (Figure 3A, Figure S4A).

**FIGURE 4:**
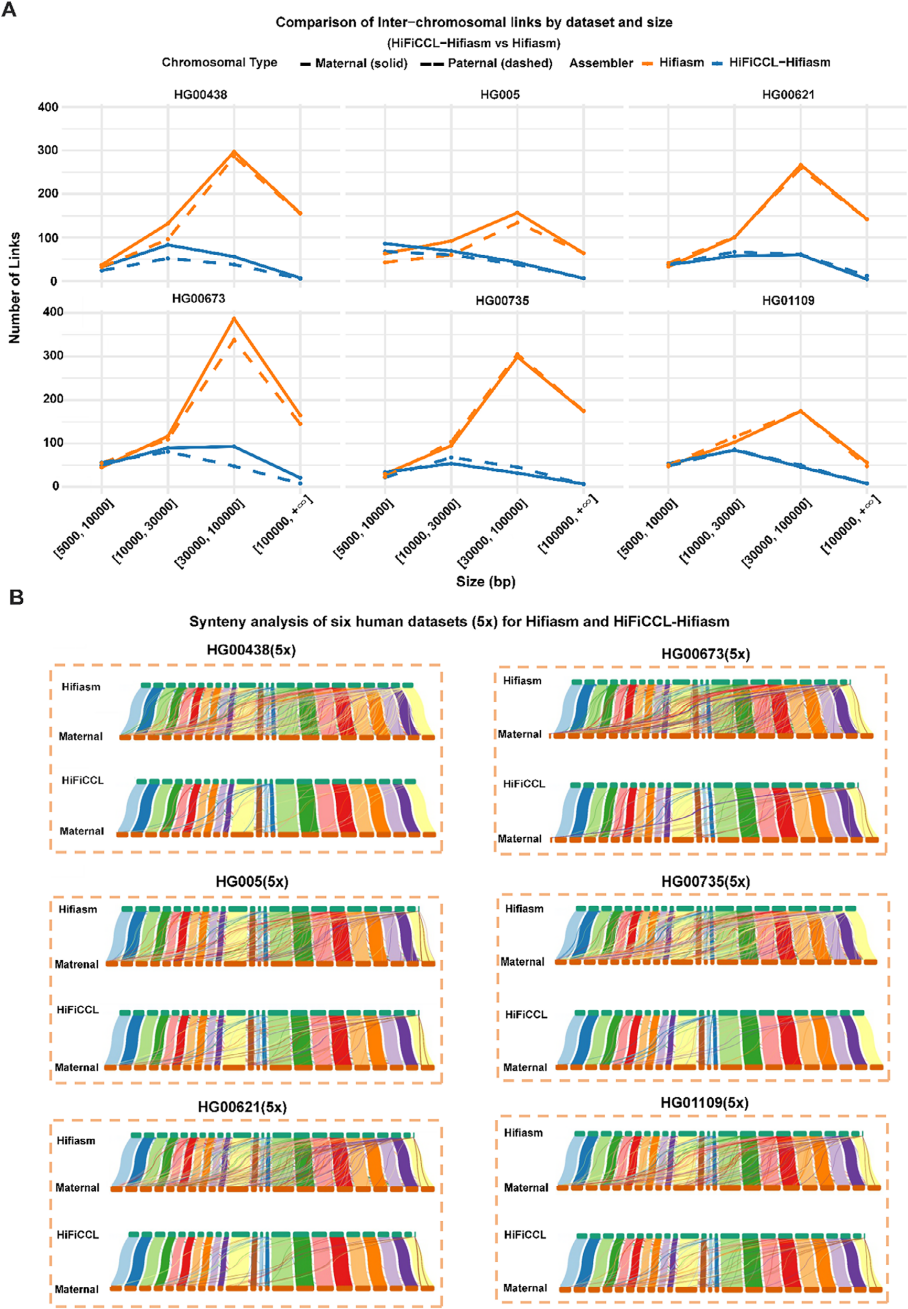
Synteny analysis of scaffolding results across multiple human datasets. (A) Synteny analysis was performed on six human datasets comparing HiFiCCL‐Hifiasm and Hifiasm post‐scaffolding, with a primary focus on the number of inter‐chromosomal links, which are considered potential erroneous translocations. (B) In each box, the top section shows the synteny analysis based on the base assembler, while the bottom section displays the synteny analysis after incorporating HiFiCCL. It shows the scaffolding performance of HiFiCCL‐Hifiasm and Hifiasm assemblies, with the maternal reference genome used as the reference, across the HG00438, HG00673, HG005, HG00735, HG00621, and HG01109 datasets (∼5×).

We further conducted a synteny analysis across six human datasets using their corresponding chromosome‐level reference assemblies, which were generated from HPRC‐released data further scaffolded with Ragtag. Four of these datasets (HG00438, HG00673, HG00735, HG00621) had previously demonstrated substantial reductions in MCL with HiFiCCL compared to Hifiasm. The remaining two datasets (HG005, HG01109), which showed only marginal improvements in earlier analyses, were included to evaluate whether HiFiCCL could achieve greater accuracy after scaffolding to the chromosome level. The results consistently demonstrated that HiFiCCL‐Hifiasm significantly reduced inter‐chromosomal mis‐scaffolding across all six datasets compared to Hifiasm (Figure 4A, Table S21). For example, in HG00438, inter‐chromosomal links in genomic regions exceeding 100 000 bp were reduced from 156 to 7 (maternal) and from 155 to 5 (paternal), representing reductions of 95.51% and 96.77%, respectively. Similarly, in HG00673, links in these regions decreased from 165 to 21 (maternal; 87.27% reduction) and from 145 to 8 (paternal; 94.48% reduction). Even in HG005, where initial improvements were modest, links exceeding 100 000 bp dropped from 64 to 6 (maternal; 90.62% reduction) and from 64 to 7 (paternal; 89.06% reduction). These results indicate that HiFiCCL can effectively reduce inter‐chromosomal read MA, thereby minimizing inter‐chromosomal mis‐scaffolding. Figure 4B and Figure S5 provide a clear visualization of how HiFiCCL effectively minimizes inter‐chromosomal mis‐scaffolding by reducing erroneous assembly of inter‐chromosomal reads.

### 2.5 Applications in Pangenomics

**FIGURE 5:**
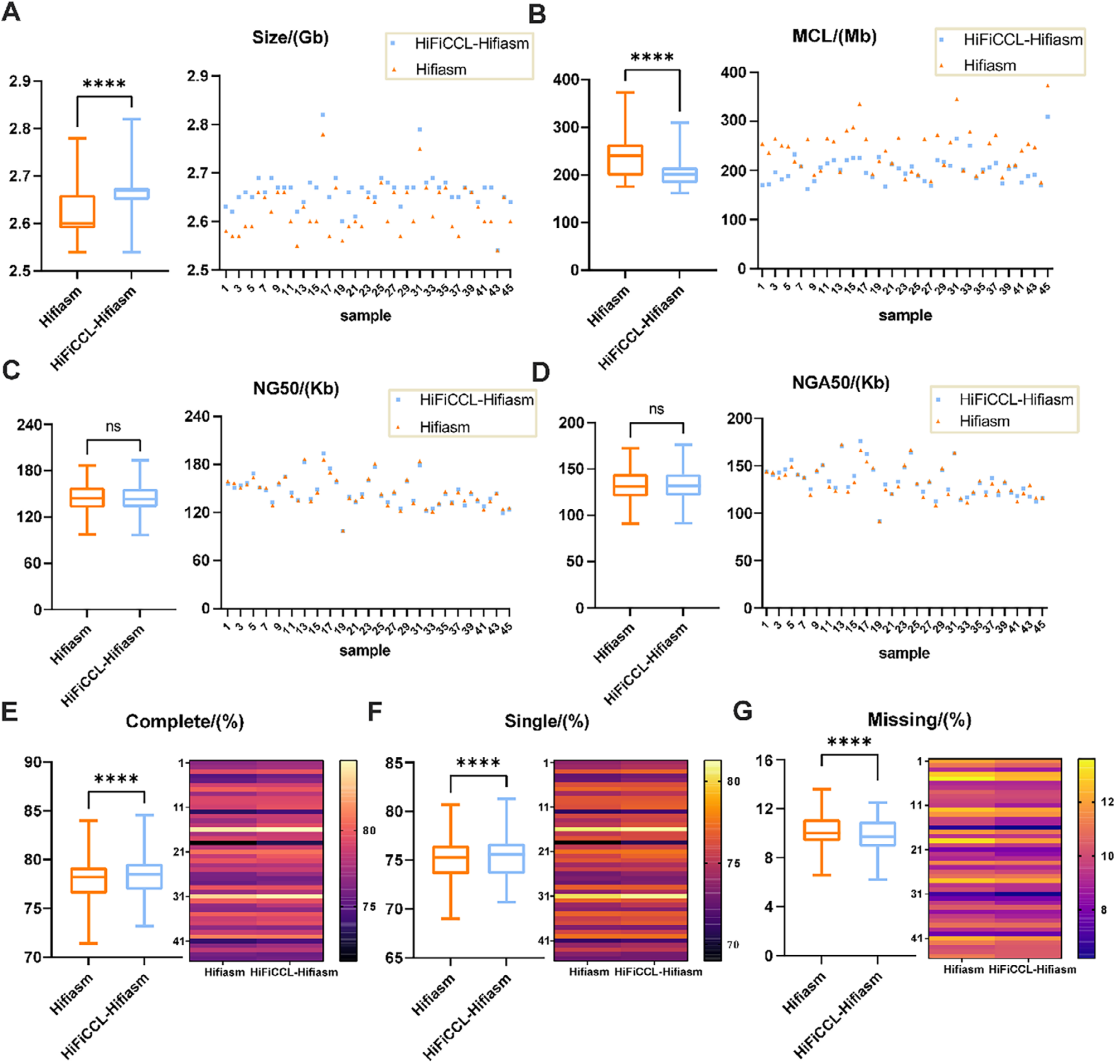
Benchmarking assemblies of Hifiasm and HiFiCCL‐Hifiasm (default mode) on 45 human samples (∼5× coverage). (A) Comparison of assembled genome size metrics between Hifiasm and HiFiCCL‐Hifiasm. (B) Comparison of misassemblies contigs length (MCL) metrics. (C) Comparison of NG50 metrics. (D) Comparison of NGA50 metrics. (E) Evaluation of gene completeness metrics. (F) Contrast of single‐copy gene completeness metrics. (G) Assessment of missing genome metrics. Statistical significance is indicated as **** for *p* < 0.0001, and ns denotes no significant difference.

To evaluate the performance of HiFiCCL in pangenome applications, we assembled genomes from 45 human low‐coverage HiFi samples (∼5×), consistent with those used in the HPRC‐released pangenome graph. Assembly accuracy was assessed using high‐quality reference genomes corresponding to the assembled samples, generated from high‐coverage sequencing data incorporating multiple data types. HiFiCCL demonstrated clear advantages in genome assembly over Hifiasm, leveraging CHM13 v2.0 as the reference genome for clustering guidance and employing its default mode to optimize assembly accuracy (Figure 5, Table S22). Compared to Hifiasm, HiFiCCL‐Hifiasm produced larger genome sizes (*p* < 0.0001) (Figure 5A) and reduced misassembled contig lengths by an average of 21.19% (up to 38.58%) (Figure 5B) while maintaining comparable contiguity (Figures 5C,D). In addition, HiFiCCL‐Hifiasm achieved significantly higher BUSCO single‐copy gene completeness (*p* < 0.0001) (Figure 5E) and a lower missing gene rate (*p* < 0.0001) (Figure 5G), showcasing its ability to generate more accurate and complete assemblies. Furthermore, we evaluated a subset of the data using Merqury [35] to assess base quality and overall completeness. The results (Table S23) demonstrated an improvement in completeness, while base quality remained comparable, with only a minimal decrease observed.

**FIGURE 6:**
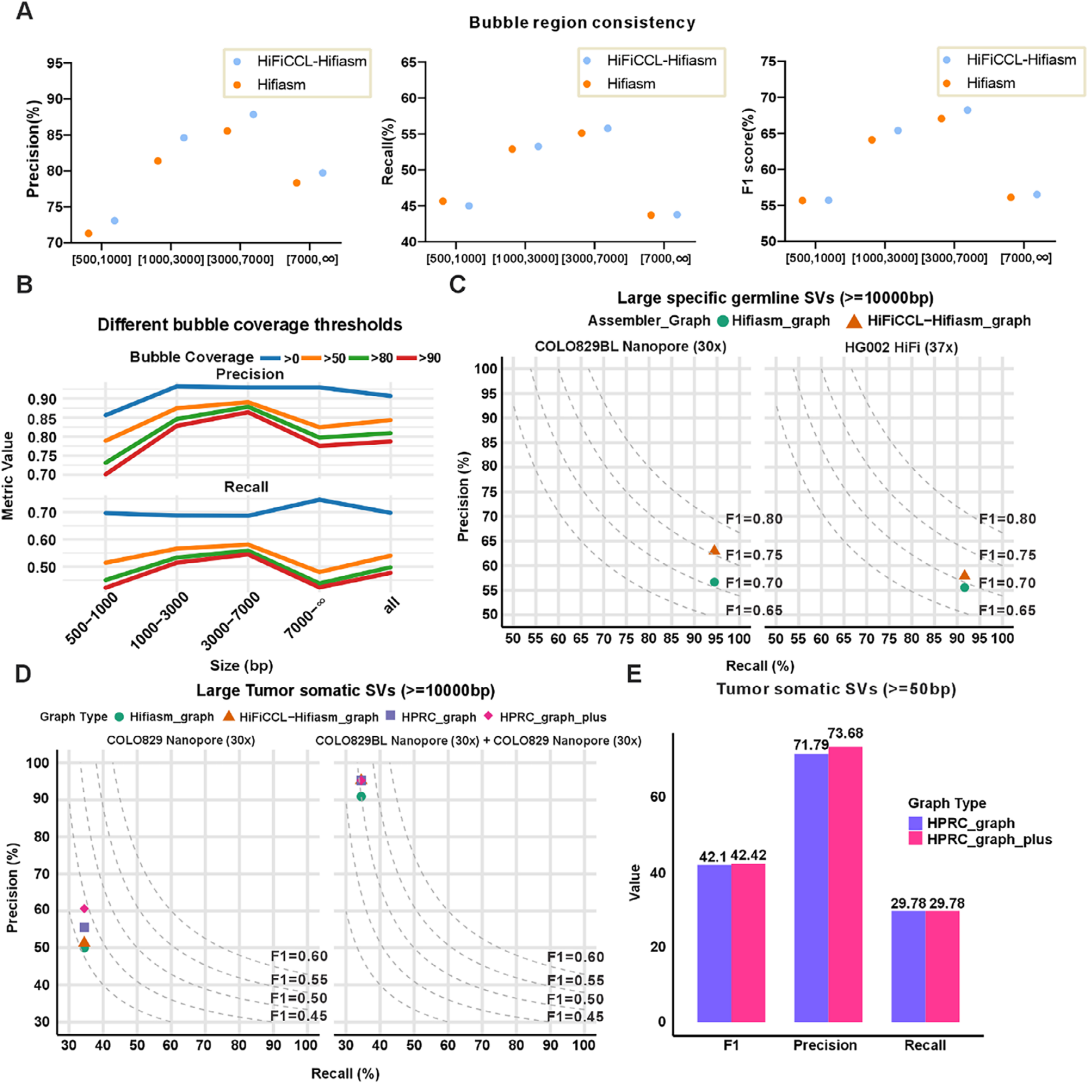
Pangenome graph consistency analysis and applications. (A) The consistency of bubble regions (bubble coverage > 80%) between pangenome graphs constructed using HiFiCCL‐Hifiasm and Hifiasm assemblies from 45 human samples with GRCh38 and CHM13 sequences against the pan‐genome graph released by the HPRC. (B) Comparison of bubble regions between pangenome graphs constructed using HiFiCCL‐Hifiasm assemblies and the pangenome graph released by the HPRC at different bubble coverage thresholds. (C) Detection of large‐specific germline SVs (≥10 000 bp) in the COLO829BL and HG002 dataset using pan‐genome graphs constructed from Hifiasm or HiFiCCL‐Hifiasm assemblies. (D) Detection of large tumor somatic structural variants (≥10 000 bp) using various pan‐genome graphs, including those constructed from assemblies generated by Hifiasm (Hifiasm_graph), HiFiCCL‐Hifiasm (HiFiCCL‐Hifiasm_graph), the pan‐genome graph released by the HPRC (HPRC_graph), and an enhanced version of the HPRC graph (HPRC_graph_plus), built using additional assemblies of 20 human genomes generated with HiFiCCL‐Hifiasm. Results are shown for COLO829 Nanopore (30×) and combined COLO829BL Nanopore (30×) + COLO829 Nanopore. (E) Detection of tumor somatic structural variants (≥50 bp) using two pan‐genome graphs: the original graph released by HPRC (HPRC_graph) and its enhanced version (HPRC_graph_plus) using the COLO829BL and COLO829 Nanopore dataset.

Using these assemblies, we constructed pangenome graphs and compared them with the real HPRC pangenome graph. HiFiCCL‐Hifiasm_graph outperformed Hifiasm_graph, showing noticeably higher precision and recall at a bubble coverage threshold of 80% (Figure 6A). Furthermore, with relaxed thresholds (e.g., >0%) (Table S24), HiFiCCL‐Hifiasm_graph achieved an overall precision of 0.9067 and recall of 0.6974, consistently maintaining superior performance across all thresholds (e.g., precision of 0.8435 and recall of 0.5399 at >50%). These results demonstrate that HiFiCCL‐Hifiasm_graph achieves higher precision and recall compared to Hifiasm_graph and aligns closely with the real HPRC pangenome graph, indicating improved accuracy and consistency in representing population genomic variation.

The constructed pangenome graphs provide a powerful tool for filtering common germline SVs, detecting large individual‐specific germline SVs (≥10 000 bp), and improving the detection of somatic SVs in both tumor‐only and tumor‐normal comparison analyses. Using the HG002 HiFi dataset (∼37×) and COLO829BL nanopore dataset (∼30×), individual‐specific germline SVs (≥10 000 bp) were detected using minisv (https://github.com/lh3/minisv) based on different pangenome graphs, including Hifiasm_graph, HiFiCCL‐Hifiasm_graph, and HPRC_graph, as well as alignments to GRCh38 and CHM13‐T2T (Figure 6C). SVs detected from HPRC_graph alignments served as the ground truth. For the HG002 dataset, HiFiCCL‐Hifiasm_graph achieved higher precision (57.89% vs. 55.55%) with the same recall (91.66%) compared to Hifiasm_graph, improving the F1 score from 68.74 to 70.96. For the COLO829BL dataset, HiFiCCL‐Hifiasm_graph improved precision (62.96% vs. 56.66%) with the same recall (94.44%), raising the F1 score from 70.83 to 75.55. These results demonstrate that HiFiCCL‐Hifiasm_graph achieves greater accuracy in representing large germline SVs.

Somatic SVs, especially large ones, are critical for understanding tumor genomics and guiding treatment strategies [48]. To evaluate the effectiveness of HiFiCCL‐Hifiasm_graph in somatic SV detection (≥10 000 bp), the COLO829 dataset (∼30×) was analyzed for somatic SV detection, and the COLO829 and COLO829BL datasets (∼30× each) were analyzed in a tumor‐normal comparison (Figure 6D). Using the COLO829 dataset alone, HiFiCCL‐Hifiasm_graph achieved higher precision (51.28% vs. 50.00%) and F1 score (41.23 vs. 40.81) than Hifiasm_graph, with the same recall (34.48%). Enhancing the HPRC_graph with 20 additional human assemblies using HiFiCCL‐Hifiasm (“HPRC_graph_plus”) further improved precision (60.60%) and F1 score (43.95%), while maintaining recall (34.48%). In the tumor‐normal comparison using COLO829 and COLO829BL datasets, HiFiCCL‐Hifiasm_graph matched the HPRC_graph in precision (95.23%), recall (34.48%), and F1 score (50.63%). For smaller somatic SVs (>50 bp), HPRC_graph_plus improved precision (73.68% vs. 71.79%) and F1 score (42.42 vs. 42.10), with the same recall (29.78%) (Figure 6E). These results validate that HiFiCCL‐Hifiasm_graph enhances the accuracy and overall performance of pangenome graphs in both germline and somatic SV detection. They also highlight the potential of HiFiCCL assemblies, including additional genomes, to improve existing pangenome graphs, enabling more comprehensive SV analyses.

## 3 Discussion

Second‐generation sequencing in population genomics is limited in its ability to resolve complex genomic regions, such as SVs and repetitive sequences. The advent of third‐generation sequencing, particularly PacBio HiFi data, has enabled the development of T2T reference sequences and pangenomes, marking a new era of genomic research with unprecedented resolution and representation of genetic diversity. Despite these advances, the high cost of long‐read sequencing compared to short‐read technologies remains a significant barrier to its application in building large‐scale population datasets (thousands of individuals or more) and studying broader eukaryotic species. Although traditional read mapping‐based SV callers remain effective for detecting many types of SVs, they are limited in their ability to detect large insertions and to reconstruct highly complex genomic regions under low‐coverage conditions [49]. For example, large insertions often result in reduced split alignments or unmapped reads when the length of the insertion exceeds the sequencing read length, leading to missed variants. Furthermore, traditional mapping approaches struggle to accurately reconstruct structurally complex regions, such as the major histocompatibility complex (MHC) and other similar highly variable regions [50]. However, existing assemblers often encounter challenges under low‐coverage conditions, where insufficient coverage can lead to erroneous assembly by merging overlapping sequences from different chromosomes. To address these challenges, we developed HiFiCCL, a reference‐guided chromosome‐by‐chromosome assembly framework that leverages high‐quality reference or pangenome graph for efficient chromosomal clustering and assembly using low‐coverage HiFi reads. HiFiCCL not only facilitates the detection of large SVs, such as insertions (>10 000 bp), but also offers the potential to guide the assembly of new species’ genomes using references from closely related species. This capability enables the generation of draft genomes at low sequencing coverage, providing a cost‐effective solution for studying non‐model organisms. In addition, HiFiCCL can reconstruct complex genomic regions, such as the MHC, with high accuracy, offering a valuable tool for population‐scale studies and comparative genomics. HiFiCCL further minimizes errors in low‐coverage assembly by employing a chromosome‐based strategy, reducing misassembly of overlapping sequences from different chromosomes. The detection of SVs, such as translocations, is achieved using long reads that span translocation breakpoints. By leveraging the translocation correction module or the *de novo* correction module, HiFiCCL integrates reads containing translocation breakpoints or other breakpoint information with reliably overlapping reads, thereby enabling the construction of highly accurate genome structures.

With the release of an increasing number of T2T and pangenome sequences for various species, HiFiCCL, leveraging its reference‐guided assembly capabilities, holds significant potential and advantages for large‐scale population genomic studies under low‐coverage conditions. This method demonstrates the potential to slightly improve genome completeness in human datasets with 5× coverage. However, due to low coverage and other limitations, many genomic regions remain missing. In the human samples tested in this study, assemblies generated using high‐coverage datasets combined with multiple data types (e.g., HG00438 requiring 41× HiFi, 44× ONT, 162× Bionano, 93× Hi‐C, and 36× second‐generation sequencing data from offspring, along with 36× and 33× second‐generation data from maternal and paternal samples, respectively [6]) achieved a high base accuracy (QV, average 53.57), exceptional completeness, and continuity. In contrast, this study utilized only low‐coverage datasets (e.g., 5× HiFi), which, while falling short of the reference genome's performance, significantly reduced assembly costs and maintained lower assembly error rates with relatively high genome completeness.

HiFiCCL has also demonstrated consistently strong performance when guided by different reference genomes for human assemblies, with closer reference genomes yielding even better performance. For plant genomes with higher genetic diversity, we have tested only two samples, highlighting the need for further testing and refinement in future studies. Unlike reference‐based strategies that rely on mapping to infer genome structure and may miss novel SVs, HiFiCCL employs a chromosome‐wise binning followed by *de novo* assembly strategy, enabling the reconstruction of new SVs. This approach ensures a more comprehensive characterization of genomic variation, particularly for previously uncharacterized or species‐specific SVs. HiFiCCL also offers the ability to process more samples with reduced costs and computational requirements, making it highly suitable for preliminary studies on population genetic structure, evolutionary relationships, and genetic diversity. Additionally, it can facilitate the screening and prioritization of samples or genomic regions for more in‐depth analysis. Moreover, its applicability extends to large‐scale low‐coverage human genome assemblies, making it a promising tool for studies investigating host genetics and gut microbial genetic diversity [51, 52, 53].

When using RagTag to scaffold assemblies, such as those generated by HiFiCCL‐Hifiasm and Hifiasm for the HG002 dataset, the scaffolding process relies on reference genome alignments, which may overlook true SVs. To mitigate this effect, we chose not to use RagTag's correct function. In a comparison of real inversion SVs on chromosome 1, the scaffolding results of HiFiCCL‐Hifiasm and Hifiasm detected only 62.5% of the variations. In addition, when we performed variant detection directly on the unscaffolded assemblies, no additional variants (compared to the true inversion SVs) were identified, likely due to insufficient sequencing coverage leading to missing region. Our future efforts will focus on integrating additional data types, such as Hi‐C or nanopore sequencing, to improve assembly completeness and accuracy under low‐coverage conditions. This integration holds the potential to further enhance chromosome‐by‐chromosome assembly performance, enabling HiFiCCL to achieve near‐complete T2T assemblies at even lower sequencing coverage, paving the way for cost‐effective and high‐precision genetic analyses. Moreover, we aim to explore the use of additional data types to refine scaffolding processes, further improving scaffolding accuracy and completeness.

## 4 Conclusion

HiFiCCL demonstrates significant improvements in assembly performance under low‐coverage conditions by leveraging high‐quality reference sequences or pangenome graphs to guide chromosome‐specific read clustering and implementing a chromosome‐by‐chromosome assembly strategy. This approach reduces inter‐chromosomal MA, improves SV detection (especially for insertions >10 000 bp), and simplifies assembly complexity. Testing across 45 low‐coverage human datasets confirmed HiFiCCL's (default mode) superior assembly quality compared to Hifiasm, with faster runtimes and comparable memory usage at ∼5× coverage. HiFiCCL offers a robust, efficient, and scalable solution for genome assembly and SV detection, making it a powerful tool for population genomics.

## 5 Experimental Section

### 5.1 Construction of alignment label graph

HiFiCCL utilizes two modes for obtaining initial chromosome classes for reads: the default linear reference genome guided mode and the optional pangenome guided mode. In the default mode, the initial chromosome classes for reads are determined by aligning sequences with the high‐quality linear reference genome using the alignment tool minimap2 [54], with parameters set to “*‐ax map‐hifi*”. In the optional mode, reads that fail to align in the primary mode are assigned chromosome classes by aligning them to the pangenome graph using minigraph [36] with parameters set to “*‐cx lr*”. Subsequently, minimap2 is used to perform inter‐sequence comparisons for all read sequences with parameters set as “*‐D –dual = no –no‐long‐join ‐k19 ‐w5 ‐U50,500 –rmq ‐A1 ‐B19 ‐O39,81 ‐E3,1 ‐H ‐e0 ‐m100 ‐N20*”. This process identifies the best alignment position for each sequence relative to others, excluding itself, and identifies multiple suboptimal alignment positions.

Based on the inter‐sequence comparison information, a bidirectional graph is constructed as shown in Figure 1. In this graph, nodes represent the IDs of each sequence, and the edges between nodes indicate the degree of overlap between sequences. The overlap degree is calculated according to Equation (1).

(1) $$\mathrm{od}=\frac{\mathrm{al}}{\mathrm{ql}}*\frac{\mathrm{al}}{\mathrm{rl}}*\frac{m}{\mathrm{al}}*w$$

where ***od*** denotes the overlap degree between sequences, ***al*** represents the alignment length between sequences, ***ql*** signifies the length of the query sequence, ***rl*** indicates the length of the reference sequence, ***m*** refers to the length of the match, and ***w*** is a constant. It is noteworthy that during the graph constructing process, if two sequences exhibit a containment relationship, the node representing the shorter sequence is collapsed into the node of the longer sequence. When analyzing inter‐sequence comparison data, if a node ***v*** align with another node ***w***, and ***v*** and ***w*** do not share a containment relationship, a directed forward edge ***α*** is established from ***v*** to ***w*** with a weight equal to the overlap degree between ***v*** and ***w***. Concurrently, a reverse edge ***β*** from ***w*** to ***v*** is constructed, carrying the same weight, representing the overlap degree between ***w*** and ***v***. Chromosome classes, derived from the alignment of sequences with the reference sequence, are assigned to each node within the graph. For sequences associated with multiple chromosome classes, all such classes are aggregated into the sequence. If a sequence fails to align to the reference genome, its chromosome class is designated as “None”. Utilizing these two types of alignment information, the construction of the final ALG is completed.

### 5.2 Construction of pangenome label graph

In the optional mode of HiFiCCL, it is necessary to realign reads that failed to align with the linear reference sequence in the primary mode to a pan‐genome graph to ascertain the chromosomal classes of these reads. Given that the chromosomal information of the linear reference genome does not coincide with that of the pan‐genome graph, a PLG is constructed to harmonize the chromosomal classes. This graph is based on the paths of the pan‐genome graph, with nodes representing the nodes of the pan‐genome graph, each tagged with its ID. Bidirectional edges are established based on the paths of the pan‐genome graph. During the alignment to the human pangenome graph, reads may exhibit classes beginning with “NA” or “HG”. These reads are processed through bidirectional DFS within the constructed PLG until chromosomal classes starting with “chr” are encountered. After standardizing the chromosomal classes, the chromosomal classes of the nodes in the ALG are assigned accordingly.

### 5.3 *De novo* class correction

Through the construction of the ALG, we have obtained a graph containing inter‐sequence alignment information along with chromosomal classes. The initial chromosomal classes of sequences are “None” if the sequences fail to align with the reference genome. In addition, similar genome regions across different chromosomes can also cause classing errors. Inspired by the Z‐drop algorithm, we propose a heuristic algorithm to search the ALG and correct sequence classes, thereby completing the classes correction of reads.

Let ***R *** = * *{***r***_1_, ***r***_2_,…, ***r***_***n***_} denote the set of reads, where **n** is the total number of reads (nodes in the graph). Let ***G*** (***V***, ***E***, ***W***) represent the ALG, where ***V*** is the set of nodes (reads). ***E*** is the set of edges representing overlaps between reads. ***W*** (***e***_***ij***_) is the weight of the edge between nodes ***i*** and ***j***, reflecting the degree of overlap between reads ***r***_***i***_ and ***r***_***j***_. Define ***C*** (***v***_***i***_) as the chromosomal class of node ***v***_***i***_ (initially assigned based on alignment to a reference genome or “None” if unclassified). A node list ***Nodelist*** is created from the set of all nodes ***V*** in the graph. The order of **Nodelist** is randomized to ensure unbiased traversal in each iteration. Nodes in **Nodelist** are traversed sequentially in the randomized order. For each node ***v***_***i***_, a bidirectional search (forward and reverse) is performed to construct a path ***PATH***_***k***_, representing a linear subgraph. The path is extended by following a set of rules: only unvisited neighboring nodes are considered for traversal, and among multiple edges, those connected via higher‐weight edges are prioritized using a greedy algorithm. If a node to be visited has already been visited, the search continues along the next priority level of edges until there are no accessible nodes left or until a node satisfies Equation (2) (indicating a significant drop in overlap degree). Nodes that do not satisfy Equation (2) are added to the path ***PATH***_***k***_. Equation (2) is described as follows:

(2) $$w_{\mathrm{jq}}\leq \theta *w_{\mathrm{ij}}+\varepsilon$$

where ***w***_***jq***_ represents the weight of the edge from the current node ***j*** to the newly accessed node ***q***, ***θ*** is a fraction less than 1, ***w***_***ij***_ denotes the weight of the edge from the previous node **i** to the current node ***j***, and ε is a constant.

For each path ***PATH***_***k***_, the chromosomal class is updated based on the majority vote among the nodes in the path:

(3) $$\mathrm{class}\_\max\left(\mathrm{PAT}H_{k}\right)=\;\mathrm{argmax}\_c\left(\mathrm{Count}\left(c,\mathrm{PAT}H_{k}\right)\right)$$

where ***Count*** (***c***, ***PATH***_***k***_) is the number of occurrences of chromosomal class ***c*** in ***PATH***_***k***_. $\mathrm{class}\_\max(\mathrm{PAT}H_{k})$ is the most frequently occurring chromosomal class in the path. If the number of nodes in the path, ***N*** (***PATH***_***k***_), satisfies the minimum support threshold $N(\mathrm{PAT}H_{k})>\mathrm{SN}\_\min$, the $\mathrm{class}\_\max(\mathrm{PAT}H_{k})$ is propagated as the updated class for all nodes in the path. All nodes in the path are marked as visited (visited = 1). The updated chromosomal classes are stored in a new hash table **NodeHash**, with the node ID as the key and its current class as the value. Once all node ***v***_***i***_ ∈ ***V*** are traversed, a single iteration is complete. The chromosomal class information from this iteration is stored in a separate hash table **ClassHash**, where:

(4) $$\mathrm{ClassHash}\;\left(v_{i}\right)=\left\{C_{1}\left(v_{i}\right),C_{2}\left(v_{i}\right),\text{…},C_{m}\left(v_{i}\right)\right\}$$

***C***_***k***_(***v***_***i***_) is the chromosomal class assigned to node ***v***_***i***_ in the ***k*** th iteration. The above process is repeated ***M*** times, with a new randomized order of **Nodelist** in each iteration. Each iteration generates a set of chromosomal class assignments for all nodes, which are recorded in **ClassHash**. After ***M*** iterations, the final chromosomal class of each node ***v***_***i***_ is determined by majority voting across all iterations:

(5) $$\mathrm{FinalClass}\;\left(v_{i}\right)=\;\mathrm{argmax}\_c\left(\mathrm{Count}\left(c,\mathrm{ClassHash}\left(v_{i}\right)\right)\right)$$

where ***Count*** (***c***, ***ClassHash*** (***v***_***i***_)) is the number of times chromosomal class ***c*** appears in the list ***ClassHash*** (***v***_***i***_) over ***M*** iterations. ***FinalClass*** (***v***_***i***_) is the most frequently occurring chromosomal class for node ***v***_***i***_. Finally, reads are clustered into chromosomal bins based on their final assigned classes:

(6) $$\mathrm{Bin}\;\left(c\right)=\{r_{i}\in R\;|\;\;\mathrm{FinalClass}\;\left(v_{i}\right)=\;c\}$$

where ***Bin*** (***c***) represents the set of reads belonging to chromosomal class ***c***.

### 5.4 Translocation class correction

Translocation variations between the input reads and the reference genome may lead to erroneous classes. Based on the alignment information with a linear reference sequence, translocation variant detection aims to identify the breakpoints where translocation occurs. This enables the correction of reads containing breakpoints information. The translocation detection module primarily employs methods like those used in the translocation detection section of CuteSV [55]. When a sequence exhibits split alignments during the matching process, each split alignment is recorded as a 6‐tuple:

(7) SI=Reads,Reade,Refs,Refe,Chr,Strand

where ***SI*** represents the split alignment information, ***Read***_***s***_ denotes the start coordinate of the aligned region in the read, ***Read***_***e***_ denotes the end coordinate of the aligned region in the read, ***Ref***_***s***_ signifies the start coordinate of the aligned region in the reference sequence, ***Ref***_***e***_ marks the end coordinate of the aligned region in the reference sequence, ***Chr*** specifies the chromosome information, and ***Strand*** indicates the direction of the alignment. If two segments of a read are aligned to different chromosomes and the distance between these segments on the sequence is less than 100 bp, their split alignment information is extracted as a translocation variant signal, as demonstrated in Equation (8).

(8) SigBND=Chr1,Ref1e,Chr2,Ref2s,ReadID,ifChr1<Chr2and++Chr2,Ref2s,Chr1,Ref1e,ReadID,ifChr2<Chr1and++Chr1,Ref1e,Chr2,Ref2e,ReadID,ifChr1<Chr2and+−Chr2,Ref2e,Chr1,Ref1e,ReadID,ifChr2<Chr1and+−Chr1,Ref1s,Chr2,Ref2s,ReadID,ifChr1<Chr2and−+Chr2,Ref2s,Chr1,Ref1s,ReadID,ifChr2<Chr1and−+Chr1,Ref1s,Chr2,Ref2e,ReadID,ifChr1<Chr2and−−Chr2,Ref2e,Chr1,Ref1s,ReadID,ifChr2<Chr1and−−

Wherein “<” indicates that the first chromosomal label is alphabetically less than the second chromosomal label, and the combinations “++”, “+‐”, “‐+”, “–” represent the orientations of SVs between chromosomes.

Subsequently, the ***Sig***_***BND***_ information is clustered by first sorting it based on genomic coordinates and creating a cluster. All signals are scanned from top to bottom to determine if they satisfy Equation (9). If a signal meets the criteria, it is added to the cluster. If it does not, a new cluster is created, and the scanning continues to assess whether additional signals can be added to an existing cluster. This process is repeated until all signals are assigned to clusters. Each cluster is then evaluated to determine if the number of signals it contains meets a predefined threshold. Clusters that do not meet this threshold are discarded. The remaining clusters are further clustered using the aforementioned method to identify three breakpoints associated with translocation variants, including two deletion breakpoints and one insertion breakpoint. Equation (9) is described as follows:

(9) $$\begin{array}{ccc} & & \left|\mathrm{Si}g_{i}\left(\mathrm{po}s_{1}\right)-\mathrm{Si}g_{j}\left(\mathrm{po}s_{1}\right)\right|\leq TH_{\mathrm{BND}}\;\mathrm{and}\;\\ & & \left|\mathrm{Si}g_{i}\left(\mathrm{po}s_{2}\right)-\mathrm{Si}g_{j}\left(\mathrm{po}s_{2}\right)\right|\leq TH_{\mathrm{BND}} \end{array}$$

where ***Sig***_***i***_(***pos***_1_) represents the prior coordinate information of signal ***i***, and ***Sig***_***j***_(***pos***_1_) represents the prior coordinate information of signal ***j***. ***TH***_***BND***_ is the predefined threshold, ***Sig***_***i***_(***pos***_2_) denotes the subsequent coordinate information of signal ***i***, and ***Sig***_***j***_(***pos***_2_) denotes the subsequent coordinate information of signal ***j***. The labels of reads carrying translocation breakpoints signals are assigned to the chromosomal information of the insertion breakpoint site and designated as enhanced nodes. This designation implies that, during de novo correction, if these reads are encountered, the classes of all reads in the path will be assigned to the chromosomal information represented by the enhanced nodes.

### 5.5 A chromosome‐by‐chromosome assembly strategy

By clustering input reads based on their respective chromosomes’ information, the original problem of assembling mixed chromosomal reads is transformed into assembling reads for each individual chromosome, significantly reducing the complexity of the original assembly process. This approach is particularly advantageous in ultra‐low‐coverage datasets, where insufficient sequence information during the path selection process can lead to MA between chromosomal sequences. Clustering reads by chromosome helps mitigate this issue by reducing the likelihood of inter‐chromosomal MA under such challenging conditions. Moreover, this allows for the parallel assembly of each chromosomal data, accelerating the assembly process. The specific workflow is as follows: First, utilize inter‐sequence alignment information and sequence‐to‐reference alignment information to construct an ALG. Next, use *de novo* class correction and translocation class correction to correct the chromosomal classes of the reads, and cluster the sequences according to their respective chromosomes. Assemble the reads for each chromosome using an existing assembler. Finally, merge the assemblies from each chromosome bin to form the final assembly.

### 5.6 Processing of reads and evaluation of assemblies

These reads were downsampled using the seqtk tool (1.3‐r106) (https://github.com/lh3/seqtk). To evaluate the assemblies, we utilized QUAST (v5.2.0) [33] to obtain basic information about the assembled contigs and assess their continuity and assembly accuracy, including contigs size, number of contigs, misassembled contigs length, as well as NG50 and NGA50 metrics. Accuracy metrics, such as MCL or the number of MA, were evaluated using high‐quality published assemblies corresponding to low‐coverage datasets. BUSCO (5.4.7) [34] was employed to assess gene completeness, using the database of conserved single copy orthologs for vertebrates in human genomes. For rice and *Arabidopsis thaliana* genome evaluation, the database of conserved single copy orthologs for angiosperms was utilized. In addition, Merqury [35] was used to evaluate assembly completeness and quality value (QV) scores, providing complementary insights into the accuracy and completeness of the genome assemblies. All experimental commands related to this study are provided in Note S3.

### 5.7 Detection and evaluation of germline SVs

SV detection based on reads alignment was performed using SVIM (2.0.0), Sniffles2 (2.4), PBSV, while assembly‐based SV detection was conducted using SVIM‐asm (1.0.3). The evaluation was carried out with Truvari (v3.2.0) [56], using GRCh37 as the reference genome.

### 5.8 Scaffolding, evaluation, and synteny analysis of assemblies

The scaffolding of different assemblies was performed using the Ragtag tool, with CHM13 (v2.0) selected as the reference genome. The evaluation of the scaffolding results was conducted using Quast and BUSCO. When using Quast for evaluation, CHM13 (v2.0) was selected as the reference genome for assessing contiguity metrics, and the reference genome for accuracy metrics was the sequence scaffolded from the high‐quality assemblies corresponding to the ultra‐low‐coverage datasets. Synteny analysis was performed using NGenomeSyn.

### 5.9 Pangenome Graph Construction and Bubble Region Consistency Evaluation

First, the downsampled HiFi (5×) datasets from 45 human samples were assembled using both HiFiCCL‐Hifiasm and Hifiasm, respectively, with each sequence renamed to ensure uniqueness across all assemblies. Then, using the GRCh38 sequence as the reference, the input data included the CHM13 (v1.0) sequence along with the HiFiCCL‐Hifiasm or Hifiasm assemblies for each sample, sorted alphabetically. These were used to construct the pangenome graph. The bubble regions of the pangenome were identified using gfatools (https://github.com/lh3/gfatools), which generated a BED file representing these regions. Using the BED file generated from the pangenome graph constructed by minigraph from the HPRC release as the reference, bubble regions in our pangenome graph with over 80% overlap with the reference were considered true positives (TP). Bubble regions that did not meet this overlap threshold were classified as false positives (FP), while bubble regions in the reference that did not overlap by at least 80% with our graph were considered false negatives (FN). The evaluation was conducted across different intervals according to the following formulas:

(10) $$Precision=\frac{TP}{TP+FP}\;$$

(11) $$Recall=TP\;/\left(TP+FN\right)$$

(12) $$F1=2\times \left(Precision\;\times Recall\right)/\left(Precision+Recall\right)$$

### 5.10 Large, specific germline SVs detection based on pangenome graph

The input dataset was first aligned to the reference genome GRCh38 using minimap2, followed by alignment to the CHM13‐T2T reference genome using the same tool. Subsequently, the input dataset was aligned to Hifiasm_graph, HiFiCCL‐Hifiasm_graph, and HPRC_graph using minigraph. The alignment results from the two linear reference genomes, as well as the alignment to Hifiasm_graph, were then input into minisv for SV detection. Similarly, SV detection results were generated by minisv based on the alignments to the different graphs. The SV detection results from the alignment to “HPRC_graph” were considered the ground truth and used to assess the performance of SV detection for variants larger than 10 000 bp, using the alignment results from “Hifiasm_graph” and “HiFiCCL_graph” as inputs.

### 5.11 Cancer somatic SVs detection based on pangenome graphs

The minisv supports two scenarios for cancer somatic SVs detection: tumor‐only and tumor‐normal paired. In the tumor‐only scenario, the tumor dataset COLO829 (30×) was aligned to the reference genomes GRCh38, CHM13‐T2T, and HPRC_graph (following the same procedure as in large, specific germline SV detection), and the results were input into minisv to obtain the SVs results using the HPRC_graph alignment. Similarly, SVs results were, respectively, obtained using the alignments to Hifiasm_graph and HiFiCCL‐Hifiasm_graph as inputs. The SVs larger than 10 000 bp were then evaluated using the COLO829 SV benchmark set. In the tumor‐normal paired scenario, the input for the three aforementioned minisv alignments was supplemented with reciprocal alignment information between the COLO829 dataset and the COLO829BL dataset (30×) using minimap2. The evaluation was conducted using the COLO829 somatic SV truth set as the benchmark [57]. Truvari was employed to compare the detected SVs against the truth set, providing precision, recall, and F1‐score metrics to assess the accuracy and performance of the SV detection.

### 5.12 Pangenome graph augmentation

Twenty human samples were assembled using HiFiCCL‐Hifiasm, and minigraph was then used to augment the “HPRC_graph”, incorporating additional variation and improving the representation of pangenomic structures.

### 5.13 Statistical analysis

We used the nonparametric Wilcoxon test for statistical analysis, where “****” indicates *p* < 0.0001, and “ns” denotes no significant difference.

## Conflicts of Interest

The authors declare no conflict of interest.

## Supporting information




**Supporting File**: advs73504‐sup‐0001‐SuppMat.docx.

## Data Availability

All data are available in the main text or the supplementary materials. The human HiFi data were obtained from the NCBI Sequence Read Archive: two runs (SRR10382244, SRR10382245) for HG002, SRR14611231 for NA19240. The CHM13v2.0 reference generated by the T2T consortium can be found at https://github.com/marbl/CHM13. The HG002 T2T reference can be found at https://github.com/marbl/hg002. The human pangenome graph constructed with CHM13+Y used as reference sequences can be found at https://github.com/human‐pangenomics/hpp_pangenome_resources?tab = readme‐ov‐file. The 45 human samples, along with 20 additional samples utilized for graph augmentation, were all sourced from the Human Pangenome Project accessible at https://s3‐us‐west‐2.amazonaws.com/human‐pangenomics/index.html?prefix = working/. The rice HiFi data (GSA: CRR573321) were obtained from the National Genomics Data Center database (NGDC) under project accession number PRJCA012143. The assembly for CX20 rice can be found under project accession PRJCA012309 in NGDC. The genome assembly for T2T‐NIP is deposited in the National Center for Biotechnology Information database under project accession number PRJNA953663 and the National Genomics Data Center database under project accession number PRJCA018610. The Arabidopsis thaliana HiFi data (GSA: CRA004538) were obtained from the NGDC. The near complete genome of Arabidopsis thaliana is available under the NGDC accession PRJCA007112. The high‐quality Arabidopsis thaliana genome assembly for assembly evaluation can be found in the Genome Warehouse at the NGDC (GWH: GWHBDNP00000000.1). For BUSCO, the embryophyta and vertebrata datasets are available at https://busco‐data.ezlab.org/v5/data/lineages/. HiFiCCL code is available at https://github.com/zjjbuqi/HiFiCCL.
