# Supplementary material for: Reference‐Guided Chromosome‐by‐Chromosome de novo Assembly at Scale Using Low‐Coverage High‐Fidelity Long‐Reads with HiFiCCL
Source: Adv Sci (Weinh). 2025 Dec 25;13(13):e15308. doi: 10.1002/advs.202515308 (PMC12955941; doi:10.1002/advs.202515308)
Supplement: Supplementary file 1 — Supporting File: advs73504‐sup‐0001‐SuppMat.docx. [file ADVS-13-e15308-s001.docx]

Supporting Information

**Reference-guided chromosome-by-chromosome *de novo* assembly at scale using low-coverage high-fidelity long-reads with HiFiCCL**

*Zhongjun Jiang1,2†, Weihua Pan3†, Runtian Gao1,2, Heng Hu1,2, Wentao Gao1,2, Murong Zhou1,2, Yu-Hang Yin1,2, Zhipeng Qian1,2, Shuilin Jin4, Guohua Wang2,5**

1 College of Life Science, Northeast Forestry University, Harbin 150000, China

2 College of Computer and Control Engineering, Northeast Forestry University, Harbin 150000, China

3 State Key Laboratory of Genome and Multi-Omics Technologies, Shenzhen Branch, Guangdong Laboratory for Lingnan Modern Agriculture, Genome Analysis Laboratory of the Ministry of Agriculture and Rural Affairs, Agricultural Genomics Institute at Shenzhen, Chinese Academy of Agricultural Sciences, Shenzhen, 518120, China

4 School of Mathematics, Harbin Institute of Technology, Harbin 150001, China

5 School of Computer Science and Technology, Harbin Institute of Technology, Harbin 150001, China

† Joint first authors

* Correspondence e-mail: [ghwang@nefu.edu.cn](mailto:ghwang@nefu.edu.cn)

**Supplementary Notes**

**Note S1.** Performance on plants datasets.

**Note S2.** Performance of HiFiCCL’s optional mode in using pangenome graphs with different tools.

**Note S3.** All the commands required to reproduce the content of this study.

**Supplementary Figures**

**Figure S1.** The whole pipeline of HiFiCCL.

**Figure. S2.** Comparison of the size of reads classified as 'None' at different coverages of the HG002 dataset.

**Figure S3.** Statistical counts of structural variations (SVs) of different sizes and types.

**Figure S4.** Synteny analysis of scaffolding results on low coverage HiFi dataset for NA19240.

**Figure S5.** Synteny analysis of scaffolding results on multiple datasets (paternal).

**Supplementary Tables**

**Table S1.** Merqury completeness and QV metrics for human primary assemblies across different assemblers.

**Table S2.** Comparison of runtime and memory usage on human datasets.

**Table S3.** Comparison of chromosomal clustering time and memory usage between HiFiCCL and GALA on human datasets.

**Table S4.** Statistics of plant primary assemblies across different assemblers.

**Table S5.** Comparison of runtime and memory usage on plant datasets.

**Table S6.** Comparison of chromosomal clustering time and memory usage between HiFiCCL and GALA on plant datasets.

**Table S7.** Statistics of human primary assemblies on the HG002 dataset at various coverage levels.

**Table S8.** Merqury completeness and QV metrics for human primary assemblies on the HG002 dataset at various coverage levels.

**Table S9.** Merqury completeness and QV metrics on the CHM13 dataset.

**Table S10.** Reconstruction performance of siamang-guided HiFiCCL-Hifiasm assembly in the MHC Region.

**Table S11.** SVs detection in HG002 GIAB Tier 1 (high-confidence regions).

**Table S12.** Comparison of large SVs (>7000bp) detection using different assembly results and read alignment on HG002 GIAB Tier 1 (high-confidence regions).

**Table S13.** Evaluation of large SV detection performance (>7,000 bp) using the latest GIAB SV benchmark dataset on HG002.

**Table S14.** Evaluation of large insertion SV detection performance (>7,000 bp) across different size ranges using the latest GIAB SV benchmark dataset on HG002.

**Table S15.** SVs detection on HG002 CMRG.

**Table S16.** Comparison of large SVs (>5000bp) detection using different assemblies and read alignment on HG002 CMRG.

**Table S17.** Statistics of human genome scaffolding across different assemblies.

**Table S18.** Statistics of chromosome-level scaffolds across different assemblies.

**Table S19.** Comparison of inversion detection across assemblers using the comprehensive inversion map.

**Table S20.** Comparison of inter-chromosomal links across different scaffoldings with the HG002 and NA19240 maternal and paternal reference genomes.

**Table S21.** Comparison of inter-chromosomal links across different scaffoldings with the maternal and paternal reference genomes of six human datasets.

**Table S22.** Statistics of human primary assemblies on the 45 human datasets (~5x).

**Table S23.** Merqury completeness and QV metrics across different human datasets.

**Table S24.** Comparison of bubble region consistency between HiFiCCL-Hifiasm pangenome graph and HPRC pangenome graph at different bubble coverage thresholds.

**Note S1.** Performance on plants datasets.

We evaluated HiFiCCL-Hifiasm on low coverage HiFi datasets for plants, targeting rice (~5x) and Arabidopsis thaliana (~5x) (Table S4). For rice, HiFiCCL-Hifiasm produced an assembly size of 321.8 Mb, closely matching the true genome size, with higher BUSCO completeness (85.4% complete, 11.4% missing) and a smaller misassembled contigs length (MCL: 38.0 Mb) compared to Hifiasm (297.8 Mb, 78.6% complete, 17.9% missing, MCL: 74.3 Mb). For Arabidopsis thaliana, HiFiCCL-Hifiasm achieved 117.6 Mb with BUSCO completeness of 87.6% complete, 10.0% missing, outperforming Hifiasm (115.9 Mb, 87.1% complete, 10.5% missing) and maintaining comparable contiguity metrics. HiFiCCL-Hifiasm demonstrated higher efficiency in runtime and memory usage compared to Hifiasm, completing assemblies faster while using comparable or less memory (Table S5). Additionally, HiFiCCL-clustering significantly outperformed GALA in both runtime and memory efficiency for both rice and Arabidopsis thaliana (Table S6). The reference genomes used by HiFiCCL and the reference genomes employed for assembly evaluation are detailed in the “Data Availability Statement.”

**Note S2.** Performance of HiFiCCL’s optional mode in using pangenome graphs with different tools.

We evaluated the optional modes of HiFiCCL using the HG002 HiFi (5×) dataset and tested pangenome graphs constructed with minigraph and minigraph-Cactus based on either CHM13 2.0 or GRCh38. The graphs are referred to as CHM13-minigraph, GRCh38-minigraph, CHM13-minigraph-cactus, and GRCh38-minigraph-cactus, all of which were released by HPRC. We compared the mapping performance of reads that failed to align to the linear reference genome CHM13 2.0 against these pan-genome graphs. Our results showed that CHM13-minigraph, GRCh38-minigraph, and CHM13-minigraph-cactus successfully aligned 16 reads, whereas GRCh38-minigraph-cactus exhibited a large number of reads classified as GRCh38#chrEBV. Furthermore, the GAF files generated from the pan-genome graphs constructed with Minigraph-Cactus did not display chromosome classifications, which are currently unsupported by the optional modes of HiFiCCL. Since the optional modes of HiFiCCL only map reads that fail to align to the linear reference genome, the impact of using pangenome graphs based on CHM13 2.0 versus GRCh38 is minimal. Additionally, in the optional modes of HiFiCCL, the differences between fine-grained graphs, such as those constructed using minigraph-Cactus, and coarse-grained graphs, such as those constructed using minigraph, remain negligible. This is because the optional mode only assigns chromosome labels based on alignment, which limits the impact of the graph granularity. Furthermore, when aligning reads originally classified as "None" against the pangenome graph, only a subset of these reads could be assigned a label. The majority of label assignments relied on *de novo* class correction (Figure S2). However, the inclusion of pangenome graph alignment improved the reliability of read class inference.

**Note S3.** All the commands required to reproduce the content of this study.

**Commands used by different assemblers and evaluation for human and plants datasets**

The reference genome used for HiFiCCL is CHM13-T2T (v2.0), while the pangenome utilized is hprc-v1.0-minigraph-chm13.gfa.

HiFiCCL: > python hificcl.py -t 20 -o <your_path> -m n -r <your_path/chm13v2.0.fa> -f <your_input.fasta> -a <assemblers>

HiFiCCL(optional)：> python hificcl.py -t 20 -o <your_path> -m p -r <your_path/chm13v2.0.fa> -R <your_path/hprc-v1.0-minigraph-chm13.gfa> -f <your_input.fasta> -a <assemblers>

Hifiasm：> hifiasm -o <your_path> -t 20 --primary <your_input.fasta>

LJA：> lja -t 20 --diploid -o <your_path> --reads <your_input.fasta>

Verkko：> verkko -d <your_path> --hifi <your_input.fasta> --threads 20

Flye: > flye --pacbio-hifi <your_input.fasta> --out-dir <your_path> --threads 20 --iterations 0

GALA: > gala <your_draft_genome> fa <your_input.fasta> pacbio-corrected --hifi -a <assemblers> -threads 20

In the case of GALA, it is necessary to specify the path to the draft genome. The draft genomes selected are the assembly results from Hifiasm, HiFlye and CHM13(2.0). The code from the original GALA paper available at https://github.com/ganlab/GALA could not be executed successfully. Instead, we used the code from https://github.com/JohnUrban/GALA for our analysis.

The commands used for the evaluation of the assembly results are as follows:

BUSCO: > busco -i <your_input.fasta> -o <your_prefix> -c 20 vertebrata_odb10 --mode genome -f --offline

For plant genomes:

BUSCO: > busco -i <your_input.fasta> -o <your_prefix> -c 20 embryophyta_odb10 --mode genome -f --offline

Quast: > python quast.py -o <your_path> -r <your_reference.fasta> -t 20 <your_input.fasta>

For evaluating basic metrics like contiguity in human datasets, the reference genome used was CHM13 (v2.0). The assembly accuracy was evaluated using reference genomes from the same sample as the assemblies. Specifically, HG002 was evaluated against the HG002-T2T reference genome, while the other human datasets were assessed using high-quality assemblies published by the HPRC. For the assembly quality assessment on rice datasets^[58]^, the reference genome used was the Nipponbare T2T genome. For the assembly quality assessment on Arabidopsis dataset^[59]^, a high-quality Arabidopsis thaliana genome^[60]^ was used as the reference.

Use Merqury to evaluate genome assemblies by first creating a k-mer database with meryl count and then running merqury.sh to generate QV, error rates, and k-mer distribution.

meryl count k=21 output <your_sample>.meryl <your_reads>.fastq

merqury.sh <your_sample>.meryl <your_assembly>.fasta <your_prefix>

**Commands used by germline SVs detection and evaluation**

Alignment-based SV detection and evaluation:

> minimap2 -ax map-hifi GRCH37.fa <input_file.fasta> -t 20 > aln.sam

> samtools view -Sb aln.sam > aln.bam

> samtools sort aln.bam -o aln_sorted.bam

> samtools index aln_sorted.bam

> svim alignment my_sample aln_sorted.bam GRCH37.fa

The SV detection algorithm based on contigs alignment was performed using svim-asm, with GRCh37 as the reference genome. The resulting files were sorted and indexed using samtools, and the “vcf” files were also sorted and indexed. The commands are as follows:

> minimap2 -a -x asm5 --cs -r2k -t 20 GRCH37.fa <your_assembly.fasta> > aln.sam

> samtools view -Sb aln.sam > aln.bam

> samtools sort aln.bam -o aln_sorted.bam

> samtools index aln_sorted.bam

> svim-asm haploid <your_dir> aln_sorted.bam GRCH37.fa

> bcftools sort <my.vcf> -o <my_sorted.vcf>

> bgzip <my_sorted.vcf>

> tabix <my_sorted.vcf.gz>

The evaluation was conducted using Truvari (v3.2.0) with the GIAB version 0.6 SV benchmark (high-confidence regions). The Challenging Medical Relevant Genes (CMRG) SV panel was also utilized for the assessment. Additionally, the latest GIAB benchmark was also included for testing. The commands are as follows:

> truvari bench --passonly -p 0 --sizefilt 50 --sizemin 50 --sizemax 1000000 --includebed HG002_SVs_Tier1_v0.6.bed -b HG002_SVs_Tier1_v0.6.vcf.gz -c <my_sorted.vcf.gz> -f hs37d5.fa -o <my_dir>

> truvari bench --passonly -p 0 --sizefilt 50 --sizemin 50 --sizemax 1000000 --includebed HG002_GRCh37_CMRG_SV_v1.00.bed -b HG002_GRCh37_CMRG_SV_v1.00.vcf.gz -c <my_sorted.vcf.gz> -f hs37d5.fa -o <my_dir>

**Commands used by scaffolding, evaluation, and synteny analysis**

The scaffolding was performed using Ragtag, with CHM13-T2T (v2.0) selected as the reference genome. The commands are as follows:

> ragtag.py scaffold <reference.fa> <query.fa>

The evaluation was similarly conducted using BUSCO and QUAST to assess basic metrics such as contiguity, with CHM13 (v2.0) selected as the reference genome. Assembly accuracy was evaluated using reference genomes consistent with the sample source of the assembled reads. Specifically, the reference genome for HG002 was HG002-T2T, while for the other human datasets, the reference genomes were the scaffolded sequences from high-quality assemblies published by HPRC, processed using Ragtag.

Synteny analysis was performed using NGenomeSyn. The commands are as follows:

> GetTwoGenomeSyn.pl -InGenomeA <your_scaffolding.fasta> - InGenomeB <your_reference.fasta> -OutPrefix <your_dir>

**Commands for pangenome graph construction and bubble region consistency evaluation**

The pangenome graph was constructed using minigraph. The shell script is as follows:

> CHM13_path="<CHM13.fasta>"

> ref=$(find . -type f -path "<GRCH38.fasta>")

> other_sample_paths=$(find . -type f -path "*/hifiasm/output_name.fasta" | sort)

> sample_paths="$CHM13_path $other_sample_paths"

> cmd=" minigraph -cxggs -t20 $ref $sample_paths > GRCH38_hifiasm_minigraph_name.gfa"

> eval $cmd

The bubble regions of the pangenome were identified using gfatools, generating a BED file:

> gfatools bubble graph.gfa > var.bed

Using the BED file generated from the pangenome graph constructed by minigraph from the HPRC release as the reference (real.bed), bubble regions in our pangenome graph with over 80% overlap with real.bed are considered true positives (TP), otherwise they are considered false positives (FP). The commands are as follows:

> bedtools intersect -a <our_graph.bed> -b real.bed -f 0.80 -u > tp.bed

> bedtools intersect -a <our_graph.bed> -b real.bed -f 0.80 -v > fp.bed

> bedtools intersect -a <real.bed> -b <our_graph.bed> -f 0.80 -v >fn.bed

The evaluation was conducted across different intervals using our provided evalu-ate_bed.py, after modifying the paths of the input files.

**Commands for large, specific germline SVs detection based on pangenome graph and evaluation**

First, the input sequence is aligned to the reference genome GRCh38 using miniamp2-2.28+, followed by alignment to the reference genome CHM13-T2T. Finally, the input is aligned using minigraph-0.21+. When the input data is HiFi data, the com-mands are as follows:

> minimap2 -cx map-hifi -s50 --ds GRCH38.fa <your_hifi_dataset.fasta> -t <threads> >hifi_grch38.paf

> minimap2 -cx map-hifi -s50 --ds CHM13-T2T.fa <your_hifi_dataset.fasta> -t <threads> >hifi_chm13.paf

> minigraph -cxlr <pangraph.gfa> <hifi_dataset.fasta> -t <threads> > hifi_pan.gaf

When the input data is nanopore data, the commands are as follows:

> minimap2 -cx map-ont GRCH38.fa <your_ont_dataset.fasta> -t <threads> > ont_grch38.paf

> minimap2 -cx map-ont CHM13-T2T.fa <ont_dataset.fasta> -t <threads> > ont_chm13.paf

> minigraph -cxlr <pangraph.gfa> <your_hifi_dataset.fasta> -t <threads> > ont_pan.gaf

Germline-specific SV detection was performed using minisv.

> minisv.js e -b data/hs38.cen-mask.bed hifi_grch38.paf hifi_chm13.paf hifi_pan.gaf | bash > hifi_sv.rsv

> cat hifi.rsv | sort -k1,1 -k2,2 -S4g | minisv.js merge - > hifi_sv.msv

> minisv.js genvcf hifi_sv.msv > hifi_sv.vcf

Similarly, ont_sv.vcf can be obtained. Evaluation was performed using Truvari, with the SV benchmark set consisting of the SVs identified by minisv from the alignments to two linear reference genomes and the pangenome graph released by HPRC (re-al.vcf). All VCF files were sorted, compressed, and indexed, followed by the evaluation.

**Commands for detecting large cancer somatic SVs**

There are two scenarios for cancer somatic variant detection using minisv: detection with only tumor samples and detection with tumor-normal pairs. For detection with only tumor samples, the process is the same as long, rare germline SV detection based on the pangenome graph. In the case of tumor-normal pairs, miniamp2 is used to perform reciprocal alignment between the tumor and normal datasets, followed by detection using minisv. The commands are as follows:

> minimap2 -x ava-ont <my_normal.fasta> <my_tumor.fasta> > normal_tumor.paf

> minisv.js e -n TUMOR -0b data/hs38.cen-mask.bed ont_grch38.paf ont_chm13.paf ont_pan.gaf normal_tumor.paf > cancer.rsv

> minisv.js extract -n Normal normal.paf > normal.rsv

> cat cancer.rsv normal.rsv | sort -k1,1 -k2,2 -S4g | minisv.js merge - | grep TUMOR | grep -v NORMAL > paired.msv

> minisv.js genvcf paired.msv > paired.vcf

The evaluation was conducted using the same parameters as mentioned above for Truvari, except that the SV benchmark set from COLO829 was selected for the evaluation. The commands used by Sniffles for detecting cancer somatic SVs are as follows:

> sniffles --input mapped_input.bam --vcf output.vcf --mosaic

**Pangenome graph augmentation commands**

The scripts for graph augmentation are as follows:

> ref=hprc-v1.0-minigraph-grch38.gfa

> other_sample_paths=$(find . -type f -path "*/hificcl_name.fasta" | sort)

> sample_paths="$other_sample_paths"

> cmd="minigraph -cxggs -t20 $ref $sample_paths > plus_minigraph.gfa"

> eval $cmd

**
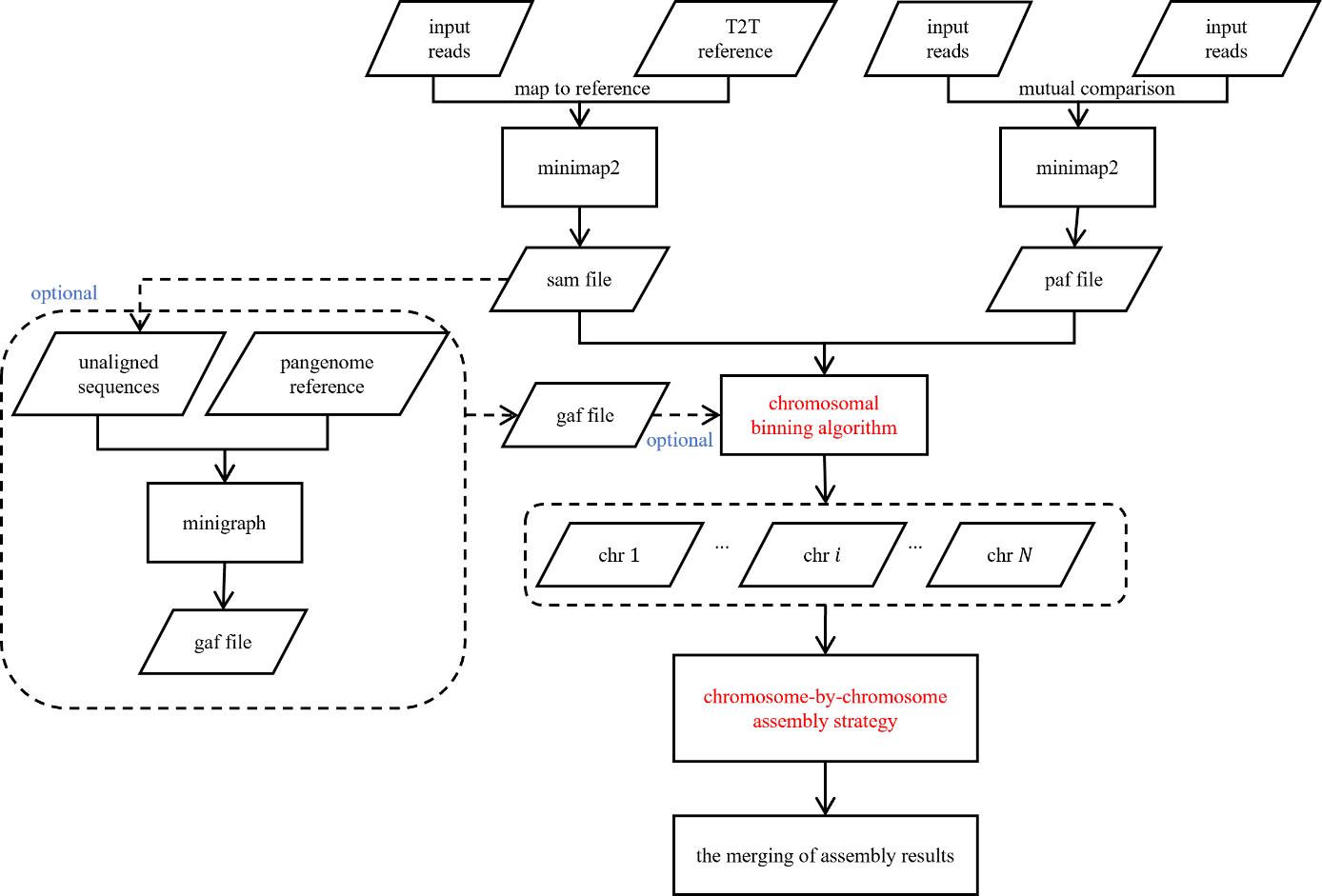
**

**Figure S1.** The whole pipeline of HiFiCCL. HiFiCCL is divided into two kinds of modes, default and optional modes. The default mode operates by utilizing alignment information between reads and the linear reference, as well as the mutual comparison information among the reads themselves to guide the binning of reads by chromosome. The optional mode, building on the default mode, further guides the binning process using alignment information of reads that did not align in the default mode with pangenome graph. Finally, the assembly is completed by employing a chromosome-by-chromosome assembly strategy.


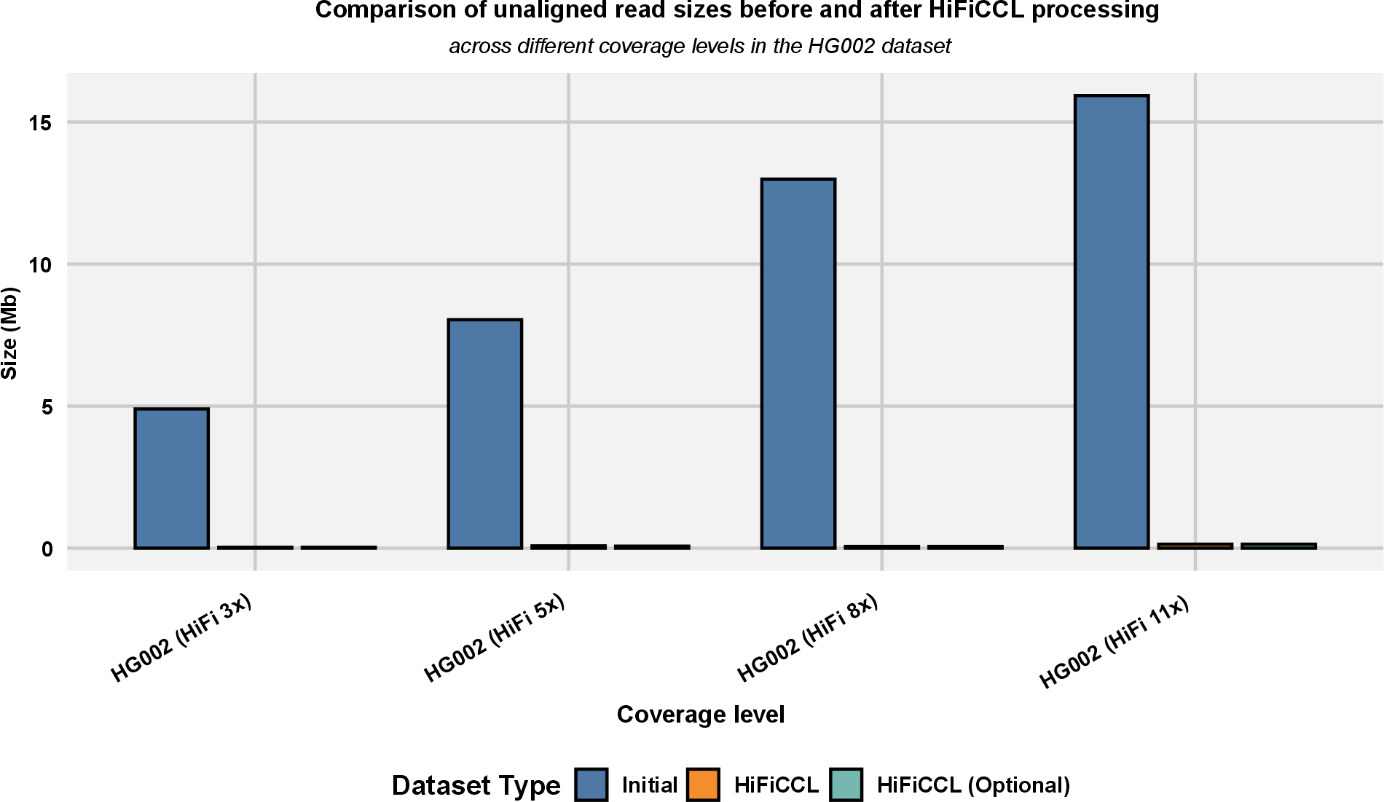
 **Figure. S2.** Comparison of the size of reads classified as 'None' at different coverages of the HG002 dataset. The "initial" category represents the size of 'None' reads directly aligned to the reference genome without HiFiCCL processing.


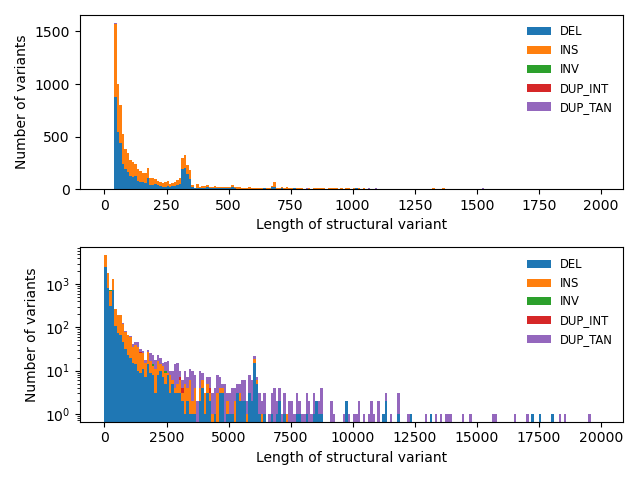

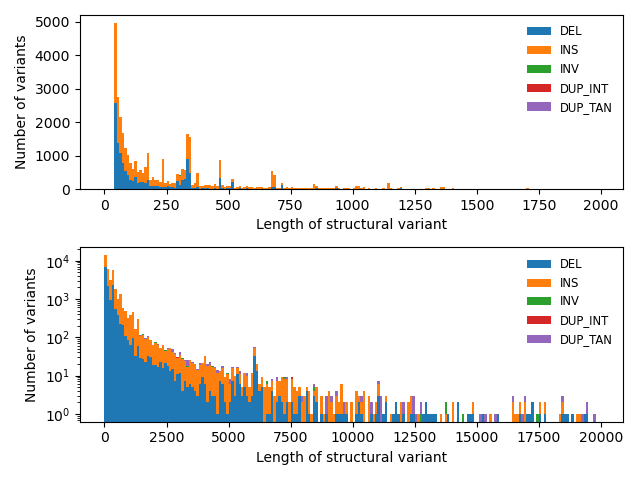

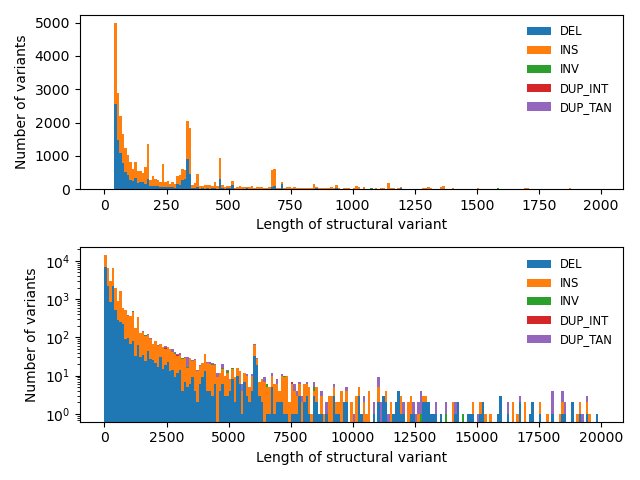

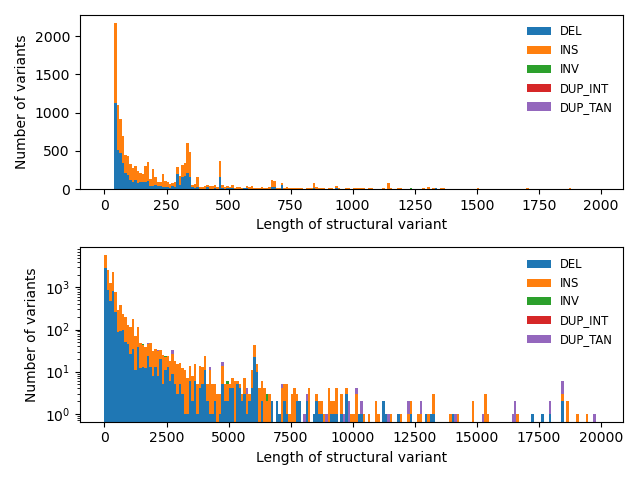

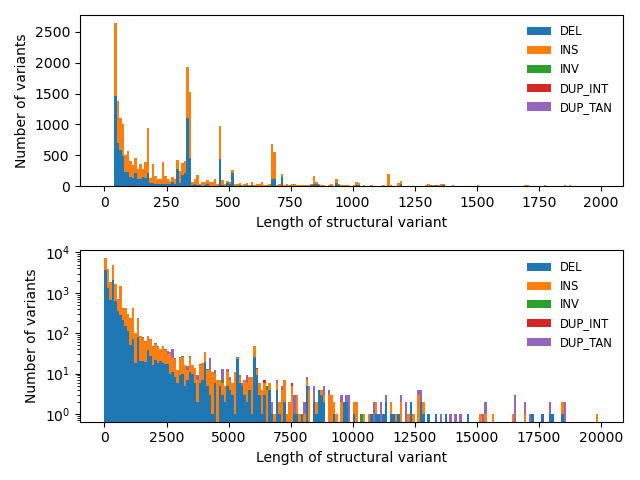

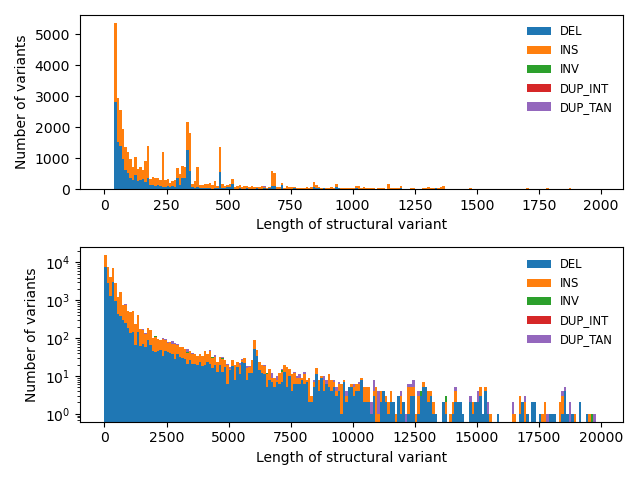


**reads**

**Hifiasm**

**HiFiCCL-Hifiasm**

**HiFlye**

**LJA**

**Verkko**

**Figure S3.** Statistical counts of structural variations (SVs) of different sizes and types. Statistics and counts of structural variations (SVs) of different sizes and types were identified based on reads-alignment detection and through alignment of assembly results from Hifiasm, HiFiCCL-Hifiasm, HiFlye, LJA, and Verkko.

**
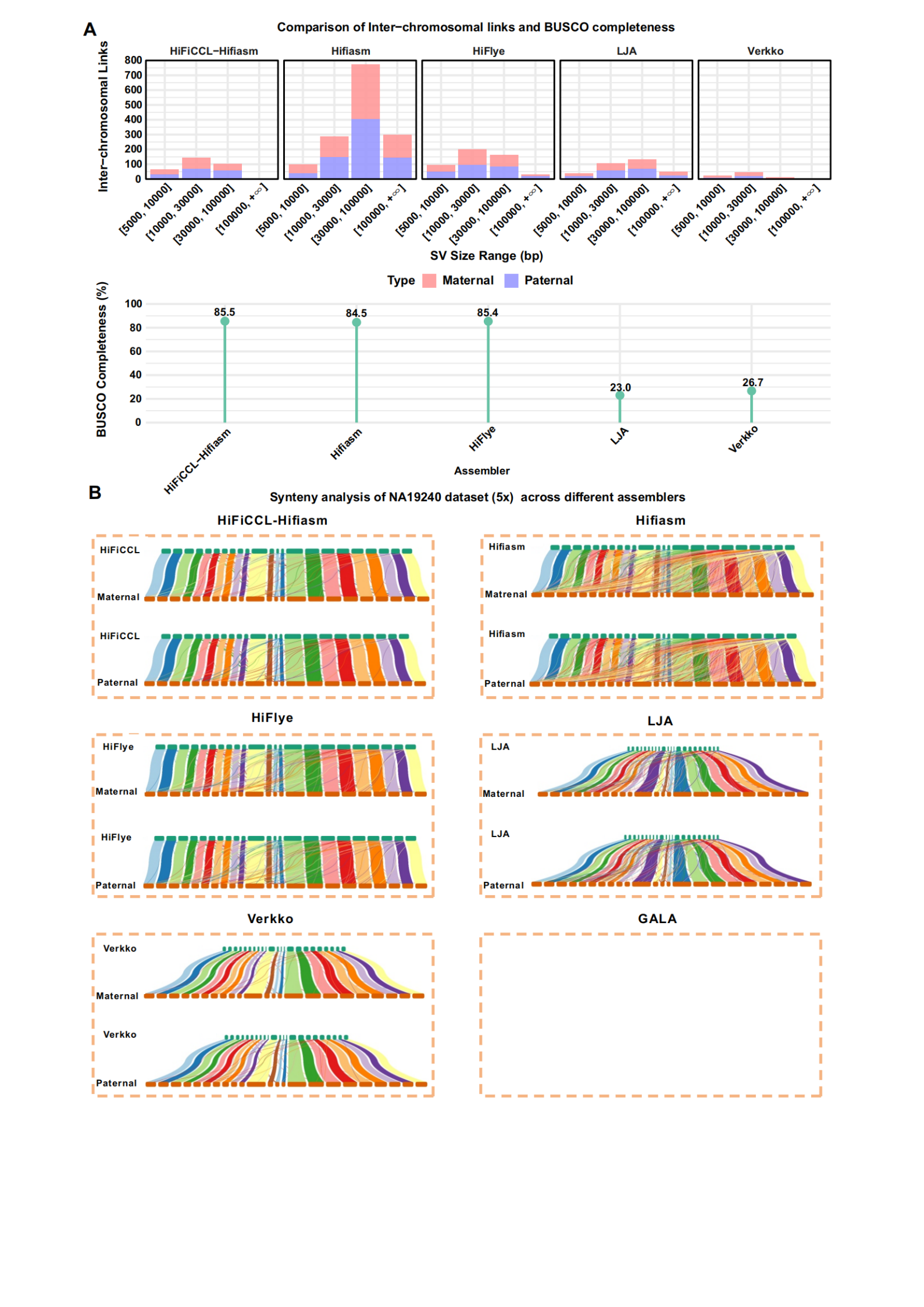
**

**Figure S4.** Synteny analysis of scaffolding results on low coverage HiFi dataset for NA19240. (A) Comparative synteny analysis of different assemblers aligned to the reference genomes of NA19240 maternal and NA19240 paternal was performed, with a primary focus on the number of inter-chromosomal links (considered as potential erroneous translocations). Additionally, the BUSCO completeness scores of assemblies generated by different assemblers were also evaluated. (B) It shows the synteny between the scaffolding performance of different assemblies and the maternal and paternal reference genomes for NA19240. Within each box, the top panel represents the synteny analysis with the maternal reference genome, while the bottom panel represents the synteny analysis with the paternal reference genome.

**
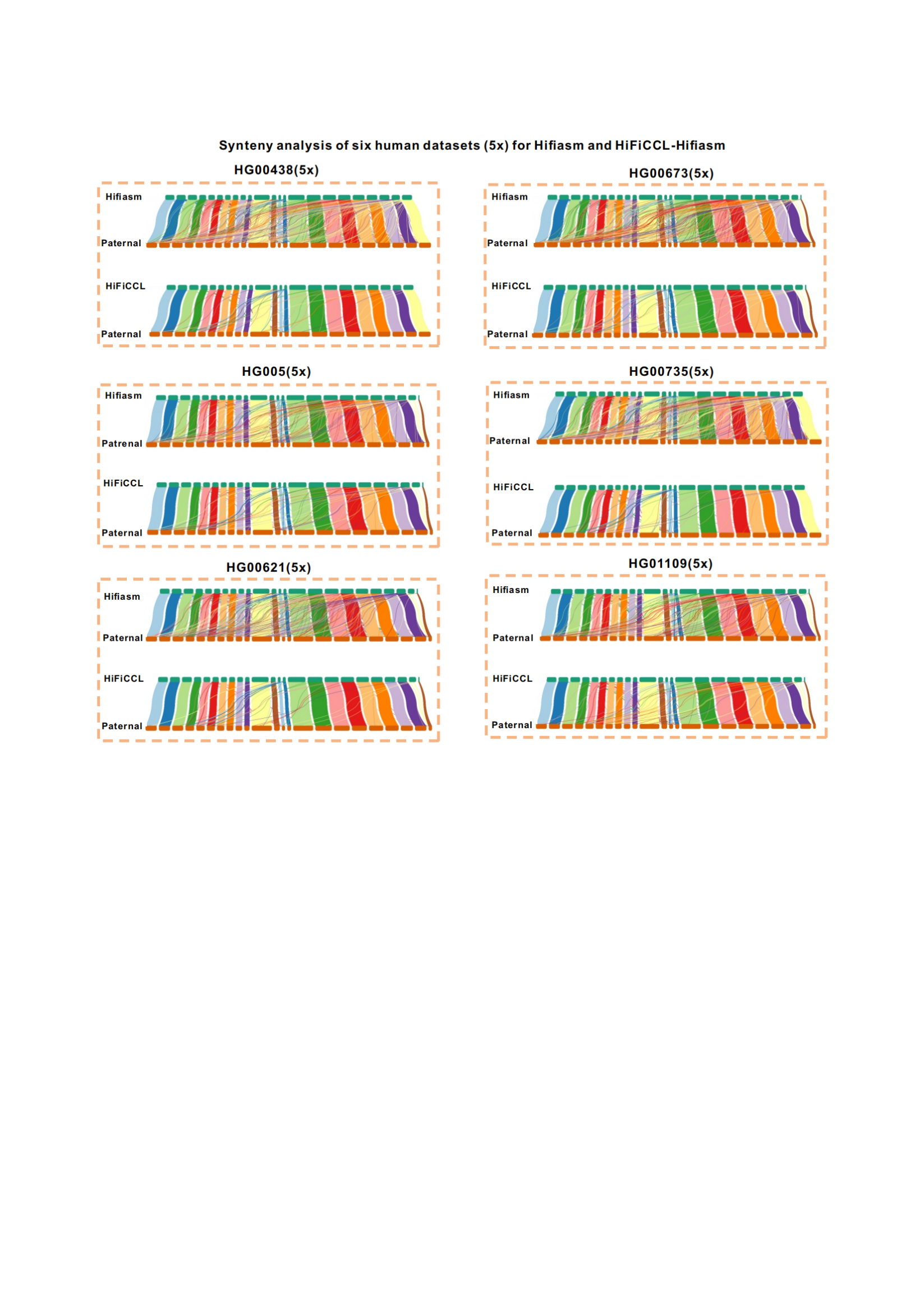
**

**Figure S5.** Synteny analysis of scaffolding results on multiple datasets (paternal). In each box, the top section shows the results based on the base assembler, while the bottom section displays the results after incorporating HiFiCCL. It shows the scaffolding performance of HiFiCCL-Hifiasm and Hifiasm assemblies, with the paternal reference genome used as the reference.

**Table S1.** Merqury completeness and QV metrics for human primary assemblies across different assemblers.

| **Dataset** | **Assembler** | **QV** | **Completeness (merqury)** |
| --- | --- | --- | --- |
| HG002  (HiFi 5x) | HiFiCCL-Hifiasm | 33.64 | 91.03 |
|  | Hifiasm | 33.72 | 89.56 |
|  | HiFlye | 34.14 | 92.56 |
|  | LJA | 31.90 | 58.64 |
|  | Verkko | 35.28 | 53.09 |
|  | GALA | - | - |
| NA19240  (HiFi 5x) | HiFiCCL-Hifiasm | 31.68 | 86.87 |
|  | Hifiasm | 31.75 | 85.26 |
|  | HiFlye | 32.55 | 86.92 |
|  | LJA | 30.79 | 21.85 |
|  | Verkko | 33.64 | 28.69 |
|  | GALA | - | - |

| **Dataset** | **Assembler** | **Elapsed (wall clock) time**  **/(h:mm:ss)** | **Maximum resident set size**  **/(kbytes)** |
| --- | --- | --- | --- |
| HG002  (5x) | HiFiCCL-Hifiasm | 5:02:09 | 55,754,616 |
|  | Hifiasm | 9:08:55 | 51,458,516 |
|  | HiFlye | 4:29:27 | 48,036,364 |
|  | LJA | 8:04:22 | 74,394,632 |
|  | Verkko | 5:27:08 | 19,012,472 |
|  | GALA | - | - |
| NA19240  (5x) | HiFiCCL-Hifiasm | 4:09:50 | 53,132,656 |
|  | Hifiasm | 6:32:53 | 51,499,152 |
|  | HiFlye | 3:43:48 | 47,933,488 |
|  | LJA | 6:24:33 | 77,701,724 |
|  | Verkko | 4:11:51 | 18,995,300 |
|  | GALA | - | - |

**Table S2.** Comparison of runtime and memory usage on human datasets.

**Table S3.** Comparison of chromosomal clustering time and memory usage between HiFiCCL and GALA on human datasets.

| **Dataset** | **Assembler** | **Elapsed (wall clock) time**  **/(h:mm:ss)** | **Maximum resident set size**  **/(kbytes)** |
| --- | --- | --- | --- |
| HG002  (5x) | HiFiCCL-clustering | 03:17:16 | 54,219,140 |
|  | GALA-clustering | 62:13:07 | 52,018,932 |
| NA19240  (5x) | HiFiCCL-clustering | 02:44:10 | 53,795,588 |
|  | GALA-clustering | 56:39:51 | 44,448,032 |

**Table S4.** Statistics of plant primary assemblies across different assemblers.

| **Dataset** | **Assembler** | **Size**  **(Mb)** | **Contigs number** | **MCL**  **(Mb)** | **NG50**  **(Kb)** | **NGA50**  **(Kb)** | **Gene completeness**  **(BUSCO)** | |
| --- | --- | --- | --- | --- | --- | --- | --- | --- |
|  | | | | | | | **Complete**  **/Single(%)** | **Missing**  **(%)** |
| Rice  (HiFi 5x) | HiFiCCL-Hifiasm | 321.8 | 5362 | 38.0 | 61.0 | 57.4 | 85.4/83.2 | 11.4 |
|  | Hifiasm | 297.8 | 3760 | 74.3 | 75.1 | 64.3 | 78.6/76.7 | 17.9 |
|  | LJA | 12.9 | 258 | 2.5 | - | - | 3.5/3.5 | 96.1 |
|  | verkko | 83.4 | 846 | 4.2 | - | - | 25.6/25.1 | 73.5 |
|  | GALA | - | - | - | - | - | - | - |
| Arabidopsisthaliana  (HiFi 5x) | HiFiCCL-Hifiasm | 117.6 | 1380 | 13.3 | 90.4 | 86.9 | 87.6/86.6 | 10.0 |
|  | Hifiasm | 115.9 | 1340 | 15.6 | 90.5 | 85.9 | 87.1/86.2 | 10.5 |
|  | LJA | - | - | - | - | - | - | - |
|  | verkko | 45.2 | 495 | 6.4 | - | - | 27.4/26.8 | 71.6 |
|  | GALA | - | - | - | - | - | - | - |

**Table S5.** Comparison of runtime and memory usage on plant datasets.

| **Dataset** | **Assembler** | **Elapsed (wall clock) time**  **/(h:mm:ss)** | **Maximum resident set size**  **/(kbytes)** |
| --- | --- | --- | --- |
| Rice  (HiFi 5x) | HiFiCCL-Hifiasm | 00:40:34 | 19,021,336 |
|  | Hifiasm | 00:20:02 | 21,519,320 |
|  | LJA | 01:05:44 | 9,723,420 |
|  | Verkko | 00:21:08 | 18,876,364 |
|  | GALA | - | - |
| Arabidopsisthaliana  (HiFi 5x) | HiFiCCL-Hifiasm | 00:12:02 | 17,425,656 |
|  | Hifiasm | 00:05:01 | 18,261,228 |
|  | LJA | 00:09:00 | 3,485,796 |
|  | Verkko | 00:14:14 | 18,391,424 |
|  | GALA | - | - |

**Table S6.** Comparison of chromosomal clustering time and memory usage between HiFiCCL and GALA on plant datasets.

| **Dataset** | **Assembler** | **Elapsed (wall clock) time**  **/(h:mm:ss)** | **Maximum resident set size**  **/(kbytes)** |
| --- | --- | --- | --- |
| Rice  (HiFi 5x) | HiFiCCL-clustering | 00:26:26 | 19,736,972 |
|  | GALA-clustering | 04:23:50 | 20,594,464 |
| Arabidopsisthaliana  (HiFi 5x) | HiFiCCL-clustering | 00:07:31 | 8,241,860 |
|  | GALA-clustering | 01:05:25 | 3,322,192 |

**Table S7.** Statistics of human primary assemblies on the HG002 dataset at various coverage levels. Bolded data indicates that the HiFiCCL metrics performance surpassed that of the base assembler, while a star in the top right corner denotes the best performance achieved.

| **Dataset** | **Assembler** | **Size**  **(Gb)** | **Contigs number** | **MCL**  **(Mb)** | **NG50**  **(Kb)** | **NGA50**  **(Kb)** | **Gene completeness**  **(busco)** | |
| --- | --- | --- | --- | --- | --- | --- | --- | --- |
|  | | | | | | | **Complete**  **/Single(%)** | **Missing**  **(%)** |
| HG002  (HiFi 3x) | Hifiasm | 2.19 | 27581 | 192.59 | 65.66 | 62.20 | 61.5/59.1 | 22.0 |
|  | HiFiCCL-Hifiasm | **2.30^*^** | 28881 | **180.93** | **69.74** | **66.28** | **63.1/60.5^*^** | **20.9** |
|  | HiFiCCL-Hifiasm  (optional) | **2.30^*^** | 28935 | **180.46^*^** | **69.78^*^** | **66.34^*^** | **63.2^*^/60.5^*^** | **20.8^*^** |
|  | HiFlye | 2.13 | 32188 | 151.06 | 56.77**^*^** | 53.22**^*^** | 54.5/51.3 | 30.1 |
|  | HiFiCCL-HiFlye | 2.12 | 32238 | **119.06** | 55.34 | 52.18 | **55.2^*^/52.2^*^** | **29.8^*^** |
|  | HiFiCCL-HiFlye)  (optional) | 2.12 | 32235 | **118.38^*^** | 55.39 | 52.27 | **55.2^*^/52.2^*^** | **29.8^*^** |
|  | LJA | - | - | - | - | - | - | - |
|  | HiFiCCL-LJA | - | - | **-** | **-** | **-** | **-** | **-** |
|  | HiFiCCL-LJA  (optional) | - | - | **-** | **-** | **-** | **-** | **-** |
| HG002  (HiFi 5x) | Hifiasm | 2.73 | 17868 | 331.84 | 194.55 | 174.55 | 82.0/78.7 | 7.6 |
|  | HiFiCCL-Hifiasm | **2.79^*^** | 18304 | **274.16** | **199.05** | **185.04** | **82.6^*^/79.1^*^** | **7.1^*^** |
|  | HiFiCCL-Hifiasm  (optional) | **2.79^*^** | 18264 | **274.07^*^** | **198.76** | **184.97** | **82.6/^*^79.0** | **7.1^*^** |
|  | HiFlye | 3.35 | 39567 | 501.71 | 199.45 | 175.02 | 84.3/76.9 | 6.1 |
|  | HiFiCCL-HiFlye | **3.34** | **39564** | **422.82** | **200.25^*^** | **177.66** | **84.8/77.1** | 6.1 |
|  | HiFiCCL-HiFlye  (optional) | **3.34** | **39417^*^** | **416.26^*^** | **199.52** | **177.76^*^** | **85.1^*^/77.8^*^** | **5.8^*^** |
|  | LJA | 1.76 | 11913 | 211.11 | 83.10 | 70.50 | 47.6/45.2 | 43.0 |
|  | HiFiCCL-LJA | 1.73 | **10753^*^** | **100.10^*^** | 81.68 | **71.54** | 47.3/45.1 | 44.6 |
|  | HiFiCCL-LJA  (optional) | 1.73 | **10792** | **101.48** | 81.88 | **71.83^*^** | 47.2/45.0 | 44.6 |
| HG002  (HiFi 8x) | Hifiasm | 3.61 | 19243 | 338.47**^*^** | 387.07 | 343.70 | 91.5/78.2 | 3.2 |
|  | HiFiCCL-Hifiasm | **3.29^*^** | **14525** | 441.76 | **459.30** | **399.82** | **91.9/83.0** | 3.2 |
|  | HiFiCCL-Hifiasm  (optional) | **3.27** | **14183^*^** | 451.49 | **464.17^*^** | **406.44^*^** | **91.9^*^/83.2^*^** | 3.2 |
|  | HiFlye | 3.89 | 46896 | 855.19 | 462.76 | 361.91 | 92.4/81.0**^*^** | 2.4 |
|  | HiFiCCL-HiFlye | 3.89 | 46988 | **832.09** | **492.60** | **388.44^*^** | **92.9^*^**/80.9 | **2.1^*^** |
|  | HiFiCCL-HiFlye  (optional) | **3.88^*^** | **46726^*^** | **831.81^*^** | **493.24^*^** | **387.55** | **92.8**/81.0**^*^** | **2.3** |
|  | LJA | 3.09 | 35718 | 120.08 | 148.37 | 138.94 | 78.7/69.4 | 11.1 |
|  | HiFiCCL-LJA | 3.11 | 36083 | **91.84** | **153.38** | **145.13** | **80.5/70.8** | **9.5** |
|  | HiFiCCL-LJA  (optional) | 3.11 | 36090 | **90.60^*^** | **153.66^*^** | **145.54^*^** | **80.7^*^/71.0^*^** | **9.3^*^** |
| HG002  (HiFi 11x) | Hifiasm | 4.74 | 21109 | 466.63***** | 605.84 | 524.14 | 96.1/59.4 | 1.4 |
|  | HiFiCCL-Hifiasm | **4.03** | **14295^*^** | 715.97 | **819.08^*^** | **688.19^*^** | 95.9/**71.0** | 1.7 |
|  | HiFiCCL-Hifiasm  (optional) | **4.07** | **14957** | 689.11 | **814.66** | **682.23** | 95.9/**71.2^*^** | 1.6 |
|  | HiFlye | 4.17 | 54508 | 971.73***** | 663.17 | 519.05 | 94.1/78.8 | 1.8 |
|  | HiFiCCL-HiFlye | 4.18 | 54972 | 981.62 | **763.90^*^** | **568.12** | **94.2***/78.8 | 2.0 |
|  | HiFiCCL-HiFlye  (optional) | 4.18 | 55006 | 978.71 | **760.06** | **573.44^*^** | 94.1/78.8 | 2.1 |
|  | LJA | 4.09 | 57160 | 106.64 | 123.48**^*^** | 116.53**^*^** | 81.2/58.2***** | 5.7 |
|  | HiFiCCL-LJA | 4.09 | 57640 | **95.12** | 121.38 | 114.48 | **81.4**/57.2 | 5.7 |
|  | HiFiCCL-LJA  (optional) | 4.08 | 57592 | **94.10*** | 121.33 | 114.52 | **81.5***/57.3 | 5.7 |

**Table S8.** Merqury completeness and QV metrics for human primary assemblies on the HG002 dataset at various coverage levels.

| **Dataset** | **Assembler** | **QV** | **Completeness (merqury)** |
| --- | --- | --- | --- |
| HG002  (HiFi 3x) | Hifiasm | 31.32 | 73.71 |
|  | HiFiCCL-Hifiasm | 31.28 | 76.53 |
|  | HiFiCCL-Hifiasm  (optional) | 31.28 | 76.62 |
|  | HiFlye | 33.25 | 67.67 |
|  | HiFiCCL-HiFlye | 33.25 | 67.36 |
|  | HiFiCCL-HiFlye)  (optional) | 33.24 | 67.38 |
|  | LJA | - | - |
|  | HiFiCCL-LJA | **-** | **-** |
|  | HiFiCCL-LJA  (optional) | **-** | **-** |
| HG002  (HiFi 5x) | Hifiasm | 33.72 | 89.56 |
|  | HiFiCCL-Hifiasm | 33.64 | 91.03 |
|  | HiFiCCL-Hifiasm  (optional) | 33.64 | 91.13 |
|  | HiFlye | 34.14 | 92.56 |
|  | HiFiCCL-HiFlye | 34.13 | 92.54 |
|  | HiFiCCL-HiFlye  (optional) | 34.13 | 92.56 |
|  | LJA | 31.90 | 58.64 |
|  | HiFiCCL-LJA | 32.87 | 58.22 |
|  | HiFiCCL-LJA  (optional) | 32.84 | 58.22 |
| HG002  (HiFi 8x) | Hifiasm | 36.46 | 95.95 |
|  | HiFiCCL-Hifiasm | 36.32 | 95.89 |
|  | HiFiCCL-Hifiasm  (optional) | 36.32 | 95.89 |
|  | HiFlye | 35.53 | 97.57 |
|  | HiFiCCL-HiFlye | 35.53 | 97.54 |
|  | HiFiCCL-HiFlye  (optional) | 39.07 | 97.54 |
|  | LJA | 34.66 | 88.15 |
|  | HiFiCCL-LJA | 35.16 | 89.06 |
|  | HiFiCCL-LJA  (optional) | 35.12 | 88.99 |
| HG002  (HiFi 11x) | Hifiasm | 38.74 | 98.44 |
|  | HiFiCCL-Hifiasm | 38.75 | 97.55 |
|  | HiFiCCL-Hifiasm  (optional) | 38.67 | 97.87 |
|  | HiFlye | 36.96 | 98.29 |
|  | HiFiCCL-HiFlye | 36.97 | 98.29 |
|  | HiFiCCL-HiFlye  (optional) | 36.97 | 98.29 |
|  | LJA | 36.69 | 95.53 |
|  | HiFiCCL-LJA | 37.01 | 95.61 |
|  | HiFiCCL-LJA  (optional) | 37.03 | 95.59 |

**Table S9.** Merqury completeness and QV metrics on the CHM13 dataset.

| **Dataset** | **Reference** | **QV** | **Complete (merqury)** |
| --- | --- | --- | --- |
| CHM13  (HiFi 5x) | - | 34.10 | 83.81 |
|  | CHM13 2.0 | 34.02 | 90.64 |
|  | HG002_maternal | 34.02 | 90.69 |
|  | HG002_paternal | 33.82 | 89.48 |
|  | hg37 | 34.07 | 90.55 |
|  | hg38 | 34.01 | 90.61 |
|  | Chimpanzee | 33.96 | 90.73 |
|  | Siamang | 33.79 | 90.49 |

**Table S10.** Reconstruction performance of siamang-guided HiFiCCL-Hifiasm assembly in the MHC Region.

| **Dataset** | **Contig** | **Gene** | **Match/Length**  **(bp)** | **Edit distance** |
| --- | --- | --- | --- | --- |
| CHM13  (HiFi 5x) | Sequence11737 | HLA-A | 3341/3341 | 0 |
|  | Sequence11740 | HLA-B | 3307/3307 | 0 |
|  | Sequence11740 | HLA-C | 3379/3379 | 0 |
|  | Sequence11922 | HLA-DQA1 | 6314/6312 | 2 |
|  | Sequence11922 | HLA-DQB1 | 7320/7320 | 0 |
|  | Sequence11922 | HLA-DRB1 | 13422/13422 | 0 |

**Table S11.** SVs detection in HG002 GIAB Tier 1 (high-confidence regions).

| **Dataset** | **Assembler** | **regions** | **Precision** | **Recall** | **F1** |
| --- | --- | --- | --- | --- | --- |
| HG002  (5x) | HiFiCCL-Hifiasm | [50, 1000] | 0.7506 | 0.6495 | 0.6964 |
|  |  | [1000, 3000] | 0.9466 | 0.6181 | 0.7479 |
|  |  | [3000, 7000] | 0.9741 | 0.5947 | 0.7385 |
|  |  | [7000, +∞] | 0.9193 | 0.5643 | 0.6993 |
|  | Hifiasm | [50, 1000] | 0.7517 | 0.6466 | 0.6952 |
|  |  | [1000, 3000] | 0.9384 | 0.5883 | 0.7232 |
|  |  | [3000, 7000] | 0.9813 | 0.5526 | 0.7070 |
|  |  | [7000, +∞] | 0.9016 | 0.5445 | 0.6790 |
| HG002 (8x) | HiFiCCL-HiFlye | [50, 1000] | 0.7440 | 0.8191 | 0.7797 |
|  |  | [1000, 3000] | 0.4849 | 0.7599 | 0.5920 |
|  |  | [3000, 7000] | 0.4065 | 0.7842 | 0.5354 |
|  |  | [7000, +∞] | 0.3148 | 0.6732 | 0.4290 |
|  | HiFlye | [50, 1000] | 0.7278 | 0.8204 | 0.7713 |
|  |  | [1000, 3000] | 0.4892 | 0.7649 | 0.5967 |
|  |  | [3000, 7000] | 0.3948 | 0.7710 | 0.5222 |
|  |  | [7000, +∞] | 0.2690 | 0.6633 | 0.3828 |
|  | HiFiCCL-LJA | [50, 1000] | 0.8492 | 0.6454 | 0.7334 |
|  |  | [1000, 3000] | 0.8833 | 0.6119 | 0.7229 |
|  |  | [3000, 7000] | 0.8686 | 0.6263 | 0.7278 |
|  |  | [7000, +∞] | 0.8382 | 0.5643 | 0.6745 |
|  | LJA | [50, 1000] | 0.8471 | 0.6312 | 0.7234 |
|  |  | [1000, 3000] | 0.8750 | 0.5833 | 0.7000 |
|  |  | [3000, 7000] | 0.8937 | 0.5973 | 0.7160 |
|  |  | [7000, +∞] | 0.8484 | 0.5544 | 0.6706 |

**Table S12.** Comparison of large SVs (>7000bp) detection using different assembly results and read alignment on HG002 GIAB Tier 1 (high-confidence regions).

| **Dataset** | **Assembler** | **Precision** | **Recall** | **F1** |
| --- | --- | --- | --- | --- |
| HG002  (5x) | reads | 0.8769 | 0.5643 | 0.6867 |
|  | Hifiasm | 0.9016 | 0.5445 | 0.6790 |
|  | HiFiCCL-Hifiasm | 0.9193 | 0.5643 | 0.6993 |
|  | HiFlye | 0.2761 | 0.6534 | 0.3882 |
|  | LJA | 0.8750 | 0.3465 | 0.4964 |
|  | Verkko | 0.7906 | 0.3366 | 0.4722 |

**Table S13.** Evaluation of large SV detection performance (>7,000 bp) using the latest GIAB SV benchmark dataset on HG002.

| **Dataset** | **SV caller** | **Assembler** | **Precision** | **Recall** | **F1** |
| --- | --- | --- | --- | --- | --- |
| HG002  (5x) | svim | _ | 0.9207 | 0.3495 | 0.5067 |
|  | pbsv | _ | 0.9144 | 0.2283 | 0.3654 |
|  | sniffle2 | _ | 0.9939 | 0.3526 | 0.5205 |
|  | svim-asm | Hifiasm | 0.9451 | 0.4190 | 0.5806 |
|  |  | HiFiCCL-Hifiasm | 0.9255 | 0.4369 | 0.5936 |
|  |  | HiFiCCL-Hifiasm (primary+alternate) | 0.9257 | 0.4621 | **0.6165** |
|  |  | HiFlye | 0.4693 | **0.5710** | 0.5152 |
|  |  | LJA | 0.9863 | 0.2866 | 0.4441 |
|  |  | Verkko | 0.9259 | 0.2594 | 0.4053 |

**Table S14.** Evaluation of large insertion SV detection performance (>7,000 bp) across different size ranges using the latest GIAB SV benchmark dataset on HG002.

| **Dataset** | **methods** | **SV Size** | **Precision** | **Recall** | **F1** |
| --- | --- | --- | --- | --- | --- |
| HG002  (5x) | svim | [7000, 10000] | 0.9996 | 0.4921 | 0.6600 |
|  |  | [10000, 15000] | 0.9986 | 0.1195 | 0.2135 |
|  |  | [15000, 20000] | 0 | 0 | 0 |
|  | pbsv | [7000, 10000] | 0.9623 | 0.4297 | 0.5941 |
|  |  | [10000, 15000] | 0.6664 | 0.0975 | 0.1701 |
|  |  | [15000, 20000] | 0 | 0 | 0 |
|  | Sniffle2 | [7000, 10000] | 0.9993 | 0.6429 | 0.7825 |
|  |  | [10000, 15000] | 0.9988 | 0.1196 | 0.2136 |
|  |  | [15000, 20000] | 0 | 0 | 0 |
|  | HiFiCCL-Hifiasm  (primary+alternate)+svim-asm | [7000, 10000] | 0.9582 | 0.6297 | 0.7600 |
|  |  | [10000, 15000] | 0.9992 | 0.4137 | 0.5851 |
|  |  | [15000, 20000] | 0.9084 | 0.4522 | 0.6038 |

**Table S15.** SVs detection on HG002 CMRG.

| **Dataset** | **Assembler** | **regions** | **Precision** | **Recall** | **F1** |
| --- | --- | --- | --- | --- | --- |
| HG002  (5x) | HiFiCCL-Hifiasm | [50, 1000] | 0.8728 | 0.5988 | 0.7103 |
|  |  | [1000, 3000] | 1.0000 | 0.5454 | 0.7058 |
|  |  | [3000, 5000] | 1.0000 | 0.2500 | 0.4000 |
|  |  | [5000, +∞] | 1.0000 | 0.6000 | 0.7499 |
|  |  | [50, +∞] | 0.8880 | 0.5862 | 0.7062 |
|  | Hifiasm | [50, 1000] | 0.8655 | 0.5988 | 0.7079 |
|  |  | [1000, 3000] | 0.9166 | 0.5000 | 0.6470 |
|  |  | [3000, 5000] | 1.0000 | 0.2500 | 0.4000 |
|  |  | [5000, +∞] | 0.7500 | 0.6000 | 0.6666 |
|  |  | [50, +∞] | 0.8676 | 0.5812 | 0.6961 |
| HG002 (8x) | HiFiCCL-HiFlye | [50, 1000] | 0.9127 | 0.7906 | 0.8473 |
|  |  | [1000, 3000] | 0.7894 | 0.6818 | 0.7317 |
|  |  | [3000, 5000] | 1.0000 | 0.5000 | 0.6666 |
|  |  | [5000, +∞] | 0.8000 | 0.8000 | 0.8000 |
|  |  | [50, +∞] | 0.8971 | 0.7733 | 0.8306 |
|  | HiFlye | [50, 1000] | 0.9078 | 0.7441 | 0.8178 |
|  |  | [1000, 3000] | 0.7647 | 0.5909 | 0.6666 |
|  |  | [3000, 5000] | 0.6000 | 0.7500 | 0.6666 |
|  |  | [5000, +∞] | 0.8000 | 0.8000 | 0.8000 |
|  |  | [50, +∞] | 0.8809 | 0.7290 | 0.7978 |
|  | HiFiCCL-LJA | [50, 1000] | 0.9270 | 0.5174 | 0.6641 |
|  |  | [1000, 3000] | 1.0000 | 0.5000 | 0.6666 |
|  |  | [3000, 5000] | 1.0000 | 0.5000 | 0.6666 |
|  |  | [5000, +∞] | 1.0000 | 0.8000 | 0.8888 |
|  |  | [50, +∞] | 0.9380 | 0.5221 | 0.6708 |
|  | LJA | [50, 1000] | 0.8541 | 0.4767 | 0.6119 |
|  |  | [1000, 3000] | 1.0000 | 0.4090 | 0.5806 |
|  |  | [3000, 5000] | 1.0000 | 0.5000 | 0.6666 |
|  |  | [5000, +∞] | 1.0000 | 0.8000 | 0.8888 |
|  |  | [50, +∞] | 0.8738 | 0.4778 | 0.6178 |

**Table S16.** Comparison of large SVs (>5000bp) detection using different assemblies and read alignment on HG002 CMRG.

| **Dataset** | **methods** | **Precision** | **Recall** | **F1** |
| --- | --- | --- | --- | --- |
| HG002  (5x) | Reads-SVIM | 0.7500 | 0.6000 | 0.6666 |
|  | Reads-sniffles2 | 1.0000 | 0.6000 | 0.7499 |
|  | Hifiasm | 0.7500 | 0.6000 | 0.6666 |
|  | HiFiCCL-Hifiasm | 1.0000 | 0.6000 | 0.7499 |

**Table S17.** Statistics of human genome scaffolding across different assemblies.

| **Dataset** | **Assembler** | **Size**  **(Gb)** | **Contigs number** | **MA** | **N50**  **(Mb)** | **NG50**  **(Mb)** | **Gene completeness**  **(busco)** | |
| --- | --- | --- | --- | --- | --- | --- | --- | --- |
|  | | | | | | | **Complete**  **/Single(%)** | **Missing**  **(%)** |
| HG002  (HiFi 5x) | HiFiCCL-Hifiasm | 2.79 | 1275 | 12822 | 136.52 | 124.67 | 89.1/85.6 | 5.8 |
|  | Hifiasm | 2.73 | 1034 | 13133 | 135.39 | 123.25 | 88.2/84.9 | 6.3 |
|  | HiFlye | 3.35 | 18315 | 17881 | 136.48 | 136.48 | 90.0/84.1 | 4.9 |
|  | LJA | 1.76 | 2620 | 7436 | 97.90 | 38.56 | 49.6/47.3 | 42.7 |
|  | Verkko | 2.02 | 8454 | 30176 | 96.15 | 52.01 | 49.2/45.9 | 42.7 |
|  | GALA | - | - | -- | - | - | - | - |
| NA19240  (HiFi 5x) | HiFiCCL-Hifiasm | 2.64 | 1172 | 15823 | 129.61 | 119.72 | 85.5/82.6 | 7.9 |
|  | Hifiasm | 2.58 | 983 | 16030 | 125.42 | 115.40 | 84.5/81.8 | 8.4 |
|  | HiFlye | 3.07 | 14194 | 23669 | 136.83 | 136.83 | 85.4/79.8 | 8.2 |
|  | LJA | 0.64 | 1126 | 3785 | 31.48 | - | 23.0/22.3 | 72.1 |
|  | Verkko | 1.06 | 5050 | 16998 | 45.20 | - | 26.7/23.2 | 63.1 |
|  | GALA | - | - | - | - | - | - | - |

**Table S18.** Statistics of chromosome-level scaffolds across different assemblies.

| **Dataset** | **Assembler** | **Size**  **(Gb)** | | **Contigs number** | **MA** | **N50**  **(Mb)** | **NG50**  **(Mb)** | **Gene completeness**  **(busco)** | | | |  |
| --- | --- | --- | --- | --- | --- | --- | --- | --- | --- | --- | --- | --- |
|  | | | | | | | | | **Complete**  **/Single(%)** | | **Missing**  **(%)** | |
| HG002  (HiFi 5x) | HiFiCCL-Hifiasm | 2.70 | 24 | | 12258 | 136.52 | 124.67 | 89.0/85.7 | | 5.8 | |  |
|  | Hifiasm | 2.65 | 24 | | 12639 | 135.39 | 123.25 | 88.2/85.0 | | 6.3 | |  |
|  | HiFlye | 2.91 | 24 | | 17290 | 147.30 | 136.48 | 89.7/86.0 | | 5.1 | |  |
|  | LJA | 1.67 | 22 | | 7270 | 97.90 | 38.56 | 49.4/47.8 | | 43.0 | |  |
|  | Verkko | 1.81 | 24 | | 29883 | 99.34 | 52.01 | 49.1/47.3 | | 42.8 | |  |
|  | GALA | - | - | | - | - | - | - | | - | |  |
| NA19240  (HiFi 5x) | HiFiCCL-Hifiasm | 2.56 | 23 | | 15294 | 129.61 | 119.72 | 85.4/82.6 | | 8.0 | |  |
|  | Hifiasm | 2.51 | 23 | | 15582 | 125.42 | 115.40 | 84.5/81.9 | | 8.4 | |  |
|  | HiFlye | 2.72 | 23 | | 23212 | 138.03 | 136.83 | 85.3/81.9 | | 8.2 | |  |
|  | LJA | 0.59 | 22 | | 3624 | 35.10 | - | 22.5/22.2 | | 72.6 | |  |
|  | Verkko | 0.93 | 23 | | 16800 | 50.03 | - | 30.3/29.4 | | 62.3 | |  |
|  | GALA | - | - | | - | - | - | - | | - | |  |

**Table S19.** Comparison of inversion detection across assemblers using the comprehensive inversion map.

| **Benchmark start** | **Benchmark end** | **Benchmark Region Size** | **Hifiasm** | **HiFiCCL** | **HiFlye** | **LJA** | **Verkko** |
| --- | --- | --- | --- | --- | --- | --- | --- |
| 13104252 | 13122521 | 18270 | √ | √ | × | × | × |
| 26641622 | 26646431 | 4810 | √ | √ | √ | × | × |
| 108310642 | 108383736 | 73095 | √ | √ | √ | × | × |
| 120299058 | 120600965 | 301908 | × | × | × | × | × |
| 144376209 | 144600799 | 224591 | √ | √ | √ | √ | × |
| 146298109 | 148672872 | 2374764 | √ | √ | √ | √ | √ |
| 149843518 | 149850293 | 6776 | × | × | × | × | × |
| 248518572 | 248529489 | 10918 | × | × | × | × | × |

**Table S20.** Comparison of inter-chromosomal links across different scaffoldings with the HG002 and NA19240 maternal and paternal reference genomes.

| **Dataset** | **Assembler** | **Size (bp)** | **Inter-chromosomal links** | |
| --- | --- | --- | --- | --- |
|  | | | **maternal** | **paternal** |
| HG002  (5x) | HiFiCCL-Hifiasm | [5000, 10000] | 81 | 64 |
|  |  | [10000, 30000] | 121 | 101 |
|  |  | [30000, 100000] | 76 | 71 |
|  |  | [100000, +∞] | 14 | 13 |
|  | Hifiasm | [5000, 10000] | 68 | 79 |
|  |  | [10000, 30000] | 137 | 154 |
|  |  | [30000, 100000] | 381 | 367 |
|  |  | [100000, +∞] | 189 | 179 |
|  | HiFlye | [5000, 10000] | 64 | 74 |
|  |  | [10000, 30000] | 193 | 195 |
|  |  | [30000, 100000] | 218 | 211 |
|  |  | [100000, +∞] | 102 | 100 |
|  | LJA | [5000, 10000] | 42 | 47 |
|  |  | [10000, 30000] | 151 | 128 |
|  |  | [30000, 100000] | 267 | 251 |
|  |  | [100000, +∞] | 124 | 134 |
|  | Verkko | [5000, 10000] | 51 | 47 |
|  |  | [10000, 30000] | 89 | 53 |
|  |  | [30000, 100000] | 46 | 42 |
|  |  | [100000, +∞] | 4 | 10 |
| NA19240(5x) | HiFiCCL-Hifiasm | [5000, 10000] | 35 | 30 |
|  |  | [10000, 30000] | 74 | 71 |
|  |  | [30000, 100000] | 42 | 59 |
|  |  | [100000, +∞] | 3 | 3 |
|  | Hifiasm | [5000, 10000] | 58 | 40 |
|  |  | [10000, 30000] | 138 | 150 |
|  |  | [30000, 100000] | 369 | 404 |
|  |  | [100000, +∞] | 155 | 143 |
|  | HiFlye | [5000, 10000] | 46 | 49 |
|  |  | [10000, 30000] | 103 | 97 |
|  |  | [30000, 100000] | 76 | 85 |
|  |  | [100000, +∞] | 14 | 19 |
|  | LJA | [5000, 10000] | 19 | 19 |
|  |  | [10000, 30000] | 48 | 58 |
|  |  | [30000, 100000] | 67 | 68 |
|  |  | [100000, +∞] | 24 | 24 |
|  | Verkko | [5000, 10000] | 14 | 9 |
|  |  | [10000, 30000] | 23 | 22 |
|  |  | [30000, 100000] | 6 | 6 |
|  |  | [100000, +∞] | 0 | 0 |

**Table S21.** Comparison of inter-chromosomal links across different scaffoldings with the maternal and paternal reference genomes of six human datasets.

| **Dataset** | **Assembler** | **Size (bp)** | **Inter-chromosomal links** | |
| --- | --- | --- | --- | --- |
|  | | | **maternal** | **paternal** |
| HG00438  (5x) | HiFiCCL-Hifiasm | [5000, 10000] | 32 | 24 |
|  |  | [10000, 30000] | 83 | 52 |
|  |  | [30000, 100000] | 56 | 38 |
|  |  | [100000, +∞] | 7 | 5 |
|  | Hifiasm | [5000, 10000] | 37 | 32 |
|  |  | [10000, 30000] | 132 | 96 |
|  |  | [30000, 100000] | 297 | 286 |
|  |  | [100000, +∞] | 156 | 155 |
| HG00673(5x) | HiFiCCL-Hifiasm | [5000, 10000] | 50 | 56 |
|  |  | [10000, 30000] | 90 | 81 |
|  |  | [30000, 100000] | 93 | 48 |
|  |  | [100000, +∞] | 21 | 8 |
|  | Hifiasm | [5000, 10000] | 45 | 55 |
|  |  | [10000, 30000] | 117 | 109 |
|  |  | [30000, 100000] | 387 | 338 |
|  |  | [100000, +∞] | 165 | 145 |
| HG005  (5x) | HiFiCCL-Hifiasm | [5000, 10000] | 86 | 68 |
|  |  | [10000, 30000] | 69 | 60 |
|  |  | [30000, 100000] | 43 | 38 |
|  |  | [100000, +∞] | 6 | 7 |
|  | Hifiasm | [5000, 10000] | 63 | 43 |
|  |  | [10000, 30000] | 92 | 60 |
|  |  | [30000, 100000] | 157 | 134 |
|  |  | [100000, +∞] | 64 | 64 |
| HG00621(5x) | HiFiCCL-Hifiasm | [5000, 10000] | 37 | 38 |
|  |  | [10000, 30000] | 58 | 67 |
|  |  | [30000, 100000] | 60 | 61 |
|  |  | [100000, +∞] | 4 | 12 |
|  | Hifiasm | [5000, 10000] | 33 | 41 |
|  |  | [10000, 30000] | 100 | 101 |
|  |  | [30000, 100000] | 267 | 259 |
|  |  | [100000, +∞] | 142 | 142 |
| HG00735(5x) | HiFiCCL-Hifiasm | [5000, 10000] | 34 | 22 |
|  |  | [10000, 30000] | 54 | 68 |
|  |  | [30000, 100000] | 32 | 46 |
|  |  | [100000, +∞] | 7 | 6 |
|  | Hifiasm | [5000, 10000] | 30 | 23 |
|  |  | [10000, 30000] | 95 | 104 |
|  |  | [30000, 100000] | 299 | 305 |
|  |  | [100000, +∞] | 174 | 175 |
| HG01109(5x) | HiFiCCL-Hifiasm | [5000, 10000] | 54 | 48 |
|  |  | [10000, 30000] | 85 | 86 |
|  |  | [30000, 100000] | 46 | 50 |
|  |  | [100000, +∞] | 8 | 8 |
|  | Hifiasm | [5000, 10000] | 48 | 50 |
|  |  | [10000, 30000] | 103 | 115 |
|  |  | [30000, 100000] | 174 | 174 |
|  |  | [100000, +∞] | 56 | 48 |

**Table S22.** Statistics of human primary assemblies on the 45 human datasets (~5x). The bold data indicates that the HiFiCCL metric’s performance surpassed that of the base assembler.

| **Dataset** | **Assembler** | **Size**  **(Gb)** | **Contigs number** | **MCL**  **(Mb)** | **NG50**  **(Kb)** | **NGA50**  **(Kb)** | **Gene completeness**  **(BUSCO)** | | | |  |
| --- | --- | --- | --- | --- | --- | --- | --- | --- | --- | --- | --- |
|  | | | | | | | | **Complete**  **/Single(%)** | | **Missing**  **(%)** | |
| HG00438 | HiFiCCL-Hifiasm | **2.63** | 18573 | **170.77** | 155.91 | 143.67 | **77.1/74.3** | | **11.5** | |  |
|  | Hifiasm | 2.58 | 17636 | 254.39 | 159.07 | 143.68 | 76.5/73.7 | | 12.1 | |  |
| HG00621 | HiFiCCL-Hifiasm | **2.62** | 19071 | **172.33** | 150.56 | 140.15 | **77.5/74.8** | | **10.6** | |  |
|  | Hifiasm | 2.57 | 17892 | 235.82 | 156.74 | 143.15 | 77.1/74.2 | | 11.1 | |  |
| HG00673 | HiFiCCL-Hifiasm | **2.65** | 19409 | **195.75** | **153.54** | **143.07** | **79.8/77.0** | | **8.8** | |  |
|  | Hifiasm | 2.57 | 18607 | 264.49 | 150.87 | 137.22 | 79.4/76.6 | | 9.3 | |  |
| HG00735 | HiFiCCL-Hifiasm | **2.66** | 18917 | **181.84** | **157.22** | **146.16** | **75.7/72.9** | | **12.3** | |  |
|  | Hifiasm | 2.59 | 18398 | 250.60 | 154.65 | 140.50 | 74.9/72.4 | | 12.8 | |  |
| HG00741 | HiFiCCL-Hifiasm | **2.65** | 17434 | **188.86** | **168.79** | **156.14** | **76.5/73.5** | | **12.5** | |  |
|  | Hifiasm | 2.59 | 17007 | 248.75 | 163.40 | 149.09 | 75.4/72.6 | | 13.6 | |  |
| HG005 | HiFiCCL-Hifiasm | **2.69** | 20947 | 233.20 | 150.82 | 140.48 | 78.9/76.3 | | 9.1 | |  |
|  | Hifiasm | 2.66 | 20475 | 218.63 | 152.20 | 140.71 | 78.9/76.3 | | 9.1 | |  |
| HG01109 | HiFiCCL-Hifiasm | **2.66** | 20199 | **207.84** | 148.04 | 136.71 | 79.8/76.8 | | **9.3** | |  |
|  | Hifiasm | 2.65 | 19782 | 208.81 | 150.43 | 137.72 | 80.1/77.1 | | 9.4 | |  |
| HG01123 | HiFiCCL-Hifiasm | **2.69** | 23569 | **161.93** | **132.59** | **125.41** | **78.2/75.1** | | **9.9** | |  |
|  | Hifiasm | 2.62 | 22901 | 263.63 | 128.85 | 119.24 | 77.2/74.4 | | 10.5 | |  |
| HG01175 | HiFiCCL-Hifiasm | **2.67** | 19451 | **178.63** | 154.43 | 143.36 | 79.2/76.4 | | **10.0** | |  |
|  | Hifiasm | 2.66 | 18951 | 190.90 | 157.58 | 145.51 | 79.2/76.5 | | 10.1 | |  |
| HG01106 | HiFiCCL-Hifiasm | **2.67** | 18653 | 206.41 | **165.14** | 150.32 | **79.3/76.7** | | **9.7** | |  |
|  | Hifiasm | 2.66 | 18319 | 199.11 | 165.10 | 151.09 | 79.2/76.5 | | 9.8 | |  |
| HG01243 | HiFiCCL-Hifiasm | **2.67** | 20978 | **213.91** | **144.72** | **133.33** | **79.6/76.9** | | **8.8** | |  |
|  | Hifiasm | 2.60 | 20312 | 265.49 | 140.78 | 127.93 | 78.8/76.2 | | 9.6 | |  |
| HG01258 | HiFiCCL-Hifiasm | **2.62** | 21108 | **220.54** | **135.51** | **126.43** | **74.3/71.6** | | **12.1** | |  |
|  | Hifiasm | 2.55 | 20182 | 259.35 | 134.56 | 123.30 | 73.6/71.0 | | 12.7 | |  |
| HG01071 | HiFiCCL-Hifiasm | **2.64** | 15953 | 200.38 | 183.15 | 170.57 | 78.5/75.9 | | **11.7** | |  |
|  | Hifiasm | 2.63 | 15640 | 197.20 | 186.67 | 172.48 | 78.5/75.9 | | 11.8 | |  |
| HG01358 | HiFiCCL-Hifiasm | **2.68** | 22168 | **221.66** | **137.07** | **127.96** | **78.3/75.6** | | **8.9** | |  |
|  | Hifiasm | 2.60 | 21303 | 281.14 | 134.13 | 122.62 | 77.1/74.5 | | 9.8 | |  |
| HG01361 | HiFiCCL-Hifiasm | **2.67** | 20495 | **225.78** | **148.69** | **139.05** | **79.5/76.3** | | **8.9** | |  |
|  | Hifiasm | 2.60 | 19830 | 287.91 | 145.15 | 132.34 | 78.3/75.2 | | 9.8 | |  |
| HG01891 | HiFiCCL-Hifiasm | **2.82** | 19157 | **225.60** | **193.65** | **176.07** | **84.6/81.3** | | **6.2** | |  |
|  | Hifiasm | 2.78 | 19091 | 335.18 | 186.14 | 166.41 | 84.0/80.7 | | 6.6 | |  |
| HG01928 | HiFiCCL-Hifiasm | **2.65** | 17093 | **194.94** | **175.16** | **162.37** | **78.5/76.0** | | **11.1** | |  |
|  | Hifiasm | 2.57 | 16546 | 263.86 | 170.02 | 154.17 | 77.6/75.2 | | 11.8 | |  |
| HG02080 | HiFiCCL-Hifiasm | **2.69** | 19363 | **187.28** | 158.06 | 145.70 | 78.8/76.1 | | 9.5 | |  |
|  | Hifiasm | 2.67 | 18863 | 192.61 | 160.53 | 147.59 | 79.2/76.5 | | 9.4 | |  |
| HG00733 | HiFiCCL-Hifiasm | **2.60** | 28231 | 226.84 | 96.63 | **91.51** | **73.2/70.7** | | **11.9** | |  |
|  | Hifiasm | 2.56 | 27106 | 218.75 | 97.21 | 91.28 | 71.4/69.0 | | 13.1 | |  |
| HG02109 | HiFiCCL-Hifiasm | **2.66** | 21222 | **167.54** | **140.04** | **129.96** | **78.7/75.8** | | **9.4** | |  |
|  | Hifiasm | 2.59 | 20539 | 240.29 | 137.81 | 125.09 | 78.1/75.3 | | 9.9 | |  |
| HG02145 | HiFiCCL-Hifiasm | **2.61** | 21147 | **212.90** | 132.77 | 120.23 | 80.7/**77.5** | | 8.0 | |  |
|  | Hifiasm | 2.60 | 20788 | 215.17 | 134.41 | 120.57 | 80.7/77.4 | | 7.9 | |  |
| HG02148 | HiFiCCL-Hifiasm | **2.67** | 21747 | **204.62** | **142.61** | **133.12** | **79.9/77.0** | | **8.4** | |  |
|  | Hifiasm | 2.59 | 20836 | 266.78 | 139.94 | 128.14 | 78.5/75.8 | | 9.4 | |  |
| HG01952 | HiFiCCL-Hifiasm | **2.66** | 18871 | 193.88 | 159.98 | 148.31 | 79.3/76.1 | | 9.2 | |  |
|  | Hifiasm | 2.65 | 18471 | 182.26 | 162.38 | 150.44 | 79.7/76.5 | | 9.0 | |  |
| HG01978 | HiFiCCL-Hifiasm | **2.65** | 16529 | 208.66 | 177.32 | 164.02 | **77.9/74.7** | | **11.1** | |  |
|  | Hifiasm | 2.64 | 16172 | 198.16 | 180.67 | 166.76 | 77.6/74.4 | | 11.4 | |  |
| HG02257 | HiFiCCL-Hifiasm | **2.69** | 21801 | **188.96** | 140.73 | 130.21 | 78.6/75.6 | | 9.1 | |  |
|  | Hifiasm | 2.68 | 21360 | 191.95 | 142.87 | 131.38 | 78.6/75.7 | | 8.9 | |  |
| HG02486 | HiFiCCL-Hifiasm | **2.68** | 22999 | **178.65** | **132.72** | **122.48** | **77.1/73.7** | | **9.9** | |  |
|  | Hifiasm | 2.60 | 22307 | 264.28 | 129.15 | 116.98 | 76.1/72.9 | | 10.4 | |  |
| HG02559 | HiFiCCL-Hifiasm | **2.67** | 20959 | **168.24** | 143.79 | 132.23 | 75.8/73.1 | | 11.1 | |  |
|  | Hifiasm | 2.66 | 20572 | 177.69 | 146.06 | 133.57 | 76.0/73.3 | | 10.9 | |  |
| HG02572 | HiFiCCL-Hifiasm | **2.63** | 22755 | **220.85** | **125.03** | **112.07** | **76.2/73.3** | | **12.0** | |  |
|  | Hifiasm | 2.57 | 22186 | 272.54 | 122.07 | 108.23 | 75.5/72.6 | | 12.6 | |  |
| HG02622 | HiFiCCL-Hifiasm | **2.67** | 18786 | 217.53 | 158.41 | 145.32 | 79.6/76.7 | | **9.3** | |  |
|  | Hifiasm | 2.66 | 18402 | 211.35 | 161.16 | 147.29 | 79.7/76.8 | | 9.4 | |  |
| HG02630 | HiFiCCL-Hifiasm | **2.67** | 22264 | **209.98** | **135.06** | **125.10** | **77.8/74.8** | | **9.3** | |  |
|  | Hifiasm | 2.60 | 21546 | 257.56 | 131.90 | 120.08 | 76.9/73.7 | | 10.0 | |  |
| HG02717 | HiFiCCL-Hifiasm | **2.79** | 20037 | **264.71** | 179.06 | 162.91 | **84.2/81.1** | | **6.3** | |  |
|  | Hifiasm | 2.75 | 18898 | 345.19 | 184.08 | 163.92 | 83.4/80.4 | | 7.0 | |  |
| HG02723 | HiFiCCL-Hifiasm | **2.68** | 25284 | 200.93 | 121.85 | 113.71 | 79.7/76.5 | | 8.0 | |  |
|  | Hifiasm | 2.67 | 24517 | 200.13 | 124.31 | 115.64 | 80.1/77.1 | | 7.8 | |  |
| HG02818 | HiFiCCL-Hifiasm | **2.69** | 24493 | **250.31** | **124.48** | **116.38** | **78.0/75.1** | | **8.7** | |  |
|  | Hifiasm | 2.61 | 23854 | 279.20 | 121.15 | 111.02 | 76.8/74.1 | | 9.6 | |  |
| HG02886 | HiFiCCL-Hifiasm | **2.67** | 23437 | **184.45** | 129.53 | 120.65 | **76.7**/73.5 | | 10.4 | |  |
|  | Hifiasm | 2.66 | 22900 | 188.71 | 131.65 | 122.40 | 76.6/73.6 | | 10.4 | |  |
| HG03453 | HiFiCCL-Hifiasm | **2.68** | 21135 | 201.41 | 143.15 | 131.87 | 79.8/76.7 | | 8.6 | |  |
|  | Hifiasm | 2.67 | 20692 | 197.39 | 145.91 | 133.94 | 80.0/76.9 | | 8.5 | |  |
| HG03486 | HiFiCCL-Hifiasm | **2.65** | 22285 | **206.87** | **132.43** | **122.00** | **79.1/76.1** | | **8.9** | |  |
|  | Hifiasm | 2.59 | 21398 | 255.42 | 132.16 | 119.23 | 78.6/75.5 | | 9.6 | |  |
| HG03492 | HiFiCCL-Hifiasm | **2.65** | 20034 | **215.16** | **148.89** | **136.81** | **79.6/76.7** | | **9.7** | |  |
|  | Hifiasm | 2.57 | 19265 | 272.54 | 145.29 | 131.21 | 79.0/76.1 | | 10.1 | |  |
| HG03516 | HiFiCCL-Hifiasm | 2.67 | 23906 | **173.34** | 128.77 | 118.77 | 76.6/73.2 | | **10.8** | |  |
|  | Hifiasm | 2.67 | 22311 | 185.85 | 134.77 | 123.60 | 76.8/73.4 | | 10.9 | |  |
| HG03540 | HiFiCCL-Hifiasm | 2.66 | 20746 | **202.87** | 142.98 | 131.83 | 78.5/75.6 | | 9.4 | |  |
|  | Hifiasm | 2.66 | 20377 | 209.93 | 145.61 | 133.25 | 78.6/75.9 | | 9.2 | |  |
| HG03579 | HiFiCCL-Hifiasm | **2.64** | 21667 | **208.55** | 134.18 | 121.89 | 81.0/77.4 | | **7.7** | |  |
|  | Hifiasm | 2.63 | 21173 | 211.46 | 136.98 | 124.07 | 81.1/77.4 | | 7.8 | |  |
| HG02055 | HiFiCCL-Hifiasm | **2.67** | 23499 | **175.22** | **127.77** | **117.86** | **74.6/71.7** | | **11.7** | |  |
|  | Hifiasm | 2.60 | 22919 | 240.66 | 123.55 | 112.26 | 73.9/71.2 | | 12.3 | |  |
| HG03098 | HiFiCCL-Hifiasm | **2.67** | 21941 | **187.67** | **137.26** | **126.09** | **77.3/74.5** | | **10.5** | |  |
|  | Hifiasm | 2.60 | 21332 | 254.50 | 134.09 | 121.13 | 76.5/74.0 | | 11.2 | |  |
| NA18906 | HiFiCCL-Hifiasm | 2.54 | 20288 | **191.55** | 128.55 | 117.74 | 78.1/75.2 | | 10.3 | |  |
|  | Hifiasm | 2.54 | 18580 | 247.60 | 144.00 | 129.59 | 78.2/75.6 | | 10.0 | |  |
| NA21309 | HiFiCCL-Hifiasm | 2.65 | 24889 | **169.35** | 119.38 | 111.97 | 75.6/73.0 | | 10.4 | |  |
|  | Hifiasm | 2.65 | 24010 | 175.45 | 124.29 | 116.15 | 76.0/73.5 | | 10.3 | |  |
| NA20129 | HiFiCCL-Hifiasm | **2.64** | 23529 | **309.73** | 124.02 | **116.01** | **75.9/72.9** | | **10.4** | |  |
|  | Hifiasm | 2.60 | 22564 | 373.13 | 125.42 | 115.87 | 75.5/72.7 | | 11.1 | |  |

**Table S23.** Merqury completeness and QV metrics across different human datasets.

| **Dataset** | **Assembler** | **QV** | **Completeness**  **(merqury)** |
| --- | --- | --- | --- |
| HG00438 | HiFiCCL-Hifiasm | 30.23 | **86.28** |
|  | Hifiasm | 30.38 | 85.17 |
| HG00621 | HiFiCCL-Hifiasm | 25.38 | **85.75** |
|  | Hifiasm | 25.48 | 80.89 |
| HG00673 | HiFiCCL-Hifiasm | 31.09 | **84.11** |
|  | Hifiasm | 31.32 | 79.68 |
| HG00735 | HiFiCCL-Hifiasm | 25.85 | **85.36** |
|  | Hifiasm | 25.90 | 83.40 |
| HG00741 | HiFiCCL-Hifiasm | 26.01 | **85.64** |
|  | Hifiasm | 26.06 | 82.95 |
| HG01109 | HiFiCCL-Hifiasm | 30.63 | **86.15** |
|  | Hifiasm | 30.68 | 86.02 |
| HG01106 | HiFiCCL-Hifiasm | 28.62 | **85.60** |
|  | Hifiasm | 28.62 | 82.94 |
| HG01243 | HiFiCCL-Hifiasm | 29.41 | **85.83** |
|  | Hifiasm | 29.41 | 85.16 |
| HG01258 | HiFiCCL-Hifiasm | 29.00 | **84.20** |
|  | Hifiasm | 31.78 | 81.75 |
| HG01358 | HiFiCCL-Hifiasm | 24.10 | **86.07** |
|  | Hifiasm | 24.12 | 84.11 |
| HG01361 | HiFiCCL-Hifiasm | 26.96 | **86.27** |
|  | Hifiasm | 26.97 | 84.57 |
| HG01928 | HiFiCCL-Hifiasm | 31.70 | **87.13** |
|  | Hifiasm | 31.77 | 85.17 |
| HG00733 | HiFiCCL-Hifiasm | 26.72 | **85.08** |
|  | Hifiasm | 26.73 | 83.01 |
| HG02145 | HiFiCCL-Hifiasm | 24.32 | **83.29** |
|  | Hifiasm | 24.32 | 82.64 |
| HG01952 | HiFiCCL-Hifiasm | 31.62 | **87.41** |
|  | Hifiasm | 31.68 | 87.32 |
| HG02257 | HiFiCCL-Hifiasm | 30.44 | 86.83 |
|  | Hifiasm | 30.47 | 87.64 |
| HG02630 | HiFiCCL-Hifiasm | 28.01 | **87.18** |
|  | Hifiasm | 28.04 | 84.35 |

**Table S24.** Comparison of bubble region consistency between HiFiCCL-Hifiasm pangenome graph and HPRC pangenome graph at different bubble coverage thresholds.

| **Assembler** | **Bubble**  **coverage (%)** | **Size (bp)** | **Precision** | **Recall** |
| --- | --- | --- | --- | --- |
| HiFiCCL-Hifiasm | >0 | 500-1000 | 0.8561 | 0.6966 |
|  |  | 1000-3000 | 0.9322 | 0.6878 |
|  |  | 3000-7000 | 0.9294 | 0.6866 |
|  |  | 7000-∞ | 0.9294 | 0.7459 |
|  |  | all | 0.9067 | 0.6974 |
| HiFiCCL-Hifiasm | >50 | 500-1000 | 0.7887 | 0.5137 |
|  |  | 1000-3000 | 0.8746 | 0.5657 |
|  |  | 3000-7000 | 0.8904 | 0.5805 |
|  |  | 7000-∞ | 0.8246 | 0.4798 |
|  |  | all | 0.8435 | 0.5399 |
| HiFiCCL-Hifiasm | >80 | 500-1000 | 0.7308 | 0.4502 |
|  |  | 1000-3000 | 0.8462 | 0.5329 |
|  |  | 3000-7000 | 0.8784 | 0.5579 |
|  |  | 7000-∞ | 0.7973 | 0.4378 |
|  |  | all | 0.8090 | 0.4974 |
| HiFiCCL-Hifiasm | >90 | 500-1000 | 0.7005 | 0.4217 |
|  |  | 1000-3000 | 0.8281 | 0.5142 |
|  |  | 3000-7000 | 0.8641 | 0.5445 |
|  |  | 7000-∞ | 0.7756 | 0.4232 |
|  |  | all | 0.7872 | 0.4769 |
